# A compute-in-memory chip based on resistive random-access memory

**DOI:** 10.1038/s41586-022-04992-8

**Published:** 2022-08-17

**Authors:** Weier Wan, Rajkumar Kubendran, Clemens Schaefer, Sukru Burc Eryilmaz, Wenqiang Zhang, Dabin Wu, Stephen Deiss, Priyanka Raina, He Qian, Bin Gao, Siddharth Joshi, Huaqiang Wu, H.-S. Philip Wong, Gert Cauwenberghs

**Affiliations:** 1 Stanford University, Stanford, CA USA; 2 University of California San Diego, La Jolla, CA USA; 3 University of Pittsburgh, Pittsburgh, PA USA; 4 University of Notre Dame, Notre Dame, IN USA; 5 Tsinghua University, Beijing, China

**Keywords:** Electrical and electronic engineering, Computer science, Electronic devices

## Abstract

Realizing increasingly complex artificial intelligence (AI) functionalities directly on edge devices calls for unprecedented energy efficiency of edge hardware. Compute-in-memory (CIM) based on resistive random-access memory (RRAM)^1^ promises to meet such demand by storing AI model weights in dense, analogue and non-volatile RRAM devices, and by performing AI computation directly within RRAM, thus eliminating power-hungry data movement between separate compute and memory^2–5^. Although recent studies have demonstrated in-memory matrix-vector multiplication on fully integrated RRAM-CIM hardware^6–17^, it remains a goal for a RRAM-CIM chip to simultaneously deliver high energy efficiency, versatility to support diverse models and software-comparable accuracy. Although efficiency, versatility and accuracy are all indispensable for broad adoption of the technology, the inter-related trade-offs among them cannot be addressed by isolated improvements on any single abstraction level of the design. Here, by co-optimizing across all hierarchies of the design from algorithms and architecture to circuits and devices, we present NeuRRAM—a RRAM-based CIM chip that simultaneously delivers versatility in reconfiguring CIM cores for diverse model architectures, energy efficiency that is two-times better than previous state-of-the-art RRAM-CIM chips across various computational bit-precisions, and inference accuracy comparable to software models quantized to four-bit weights across various AI tasks, including accuracy of 99.0 percent on MNIST^18^ and 85.7 percent on CIFAR-10^19^ image classification, 84.7-percent accuracy on Google speech command recognition^20^, and a 70-percent reduction in image-reconstruction error on a Bayesian image-recovery task.

## Abstract (web summary)

A compute-in-memory neural-network inference accelerator based on resistive random-access memory simultaneously improves energy efficiency, flexibility and accuracy compared with existing hardware by co-optimizing across all hierarchies of the design.

## Main

Early research in the area of resistive random-access memory (RRAM) compute-in-memory (CIM) focused on demonstrating artificial intelligence (AI) functionalities on fabricated RRAM devices while using off-chip software and hardware to implement essential functionalities such as analogue-to-digital conversion and neuron activations for a complete system^2,3,6,20–27^. Although these studies proposed various techniques to mitigate the impacts of analogue-related hardware non-idealities on inference accuracy, the AI benchmark results reported were often obtained by performing software emulation based on characterized device data^3,5,21,24^. Such an approach often overestimates accuracies compared with fully hardware-measured results owing to incomplete modelling of hardware non-idealities.

More recent studies have demonstrated fully integrated RRAM complementary metal–oxide–semiconductor (CMOS) chips capable of performing in-memory matrix-vector multiplication (MVM)^6–17^. However, for a RRAM-CIM chip to be broadly adopted in practical AI applications, it needs to simultaneously deliver high energy efficiency, the flexibility to support diverse AI model architectures and software-comparable inference accuracy. So far, there has not been a study aimed at simultaneously improving all these three aspects of a design. Moreover, AI application-level benchmarks in previous studies have limited diversity and complexity. None of the studies have experimentally measured multiple edge AI applications with complexity matching those in MLPerf Tiny, a commonly used benchmark suite for edge AI hardware^28^. The challenge arises from the inter-related trade-offs between efficiency, flexibility and accuracy. The highly-parallel analogue computation within RRAM-CIM architecture brings superior efficiency, but makes it challenging to realize the same level of functional flexibility and computational accuracy as in digital circuits. Meanwhile, attaining algorithmic resiliency to hardware non-idealities becomes more difficult for more complex AI tasks owing to using less over-parameterized models on the edge^29,30^.

**Fig. 1:**
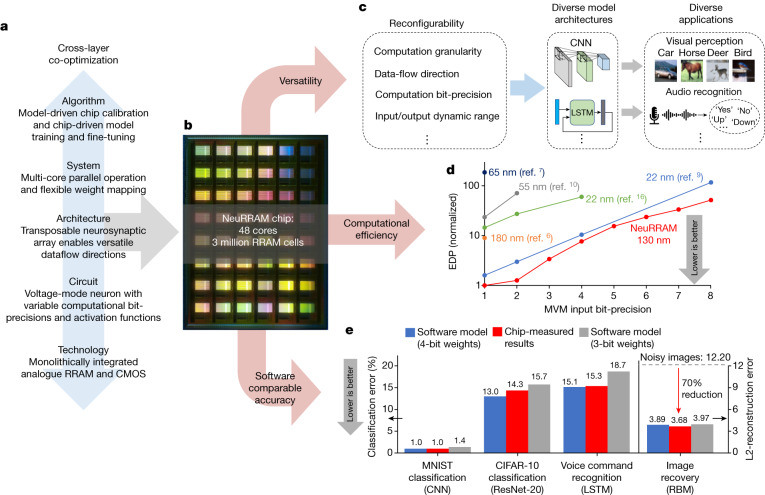
Design methodology and main contributions of the NeuRRAM chip. **a**, Cross-layer co-optimizations across the full stack of the design enable NeuRRAM to simultaneously deliver high versatility, computational efficiency and software-comparable inference accuracy. **b**, Micrograph of the NeuRRAM chip. **c**, Reconfigurability in various aspects of the design enables NeuRRAM to implement diverse AI models for a wide variety of applications. **d**, Comparison of EDP, a commonly used energy-efficiency and performance metric among recent RRAM-based CIM hardware. **e**, Fully hardware-measured inference accuracy on NeuRRAM is comparable to software models quantized to 4-bit weights across various AI benchmarks.

To address these challenges, we present NeuRRAM, a 48-core RRAM-CIM hardware encompassing innovations across the full stack of the design. (1) At the device level, 3 million RRAM devices with high analogue programmability are monolithically integrated with CMOS circuits. (2) At the circuit level, a voltage-mode neuron circuit supports variable computation bit-precision and activation functions while performing analogue-to-digital conversion at low power consumption and compact-area footprint. (3) At the architecture level, a bidirectional transposable neurosynaptic array (TNSA) architecture enables reconfigurability in dataflow directions with minimal area and energy overheads. (4) At the system level, 48 CIM cores can perform inference in parallel and supports various weight-mapping strategies. (5) Finally, at the algorithm level, various hardware-algorithm co-optimization techniques mitigate the impact of hardware non-idealities on inference accuracy. We report fully hardware-measured inference results for a range of AI tasks including image classifications using CIFAR-10^19^ and MNIST^18^ datasets, Google speech command recognition^20^ and MNIST image recovery, implemented with diverse AI models including convolutional neural networks (CNNs)^31^, long short-term memory (LSTM)^32^ and probabilistic graphical models^33^ (Fig. 1e). The chip is measured to achieve an energy-delay product (EDP) lower than previous state-of-the-art RRAM-CIM chips, while it operates over a range of configurations to suit various AI benchmark applications (Fig. 1d).

## Reconfigurable RRAM-CIM architecture

A NeuRRAM chip consists of 48 CIM cores that can perform computation in parallel. A core can be selectively turned off through power gating when not actively used, whereas the model weights are retained by the non-volatile RRAM devices. Central to each core is a TNSA consisting of 256 × 256 RRAM cells and 256 CMOS neuron circuits that implement analogue-to-digital converters (ADCs) and activation functions. Additional peripheral circuits along the edge provides inference control and manages RRAM programming.

The TNSA architecture is designed to offer flexible control of dataflow directions, which is crucial for enabling diverse model architectures with different dataflow patterns. For instance, in CNNs that are commonly applied to vision-related tasks, data flows in a single direction through layers to generate data representations at different abstraction levels; in LSTMs that are used to process temporal data such as audio signals, data travel recurrently through the same layer for multiple time steps; in probabilistic graphical models such as a restricted Boltzmann machine (RBM), probabilistic sampling is performed back and forth between layers until the network converges to a high-probability state. Besides inference, the error back-propagation during gradient-descent training of multiple AI models requires reversing the direction of dataflow through the network.

However, conventional RRAM-CIM architectures are limited to perform MVM in a single direction by hardwiring rows and columns of the RRAM crossbar array to dedicated circuits on the periphery to drive inputs and measure outputs. Some studies implement reconfigurable dataflow directions by adding extra hardware, which incurs substantial energy, latency and area penalties (Extended Data Fig. 2): executing bidirectional (forwards and backwards) dataflow requires either duplicating power-hungry and area-hungry ADCs at both ends of the RRAM array^11,34^ or dedicating a large area to routing both rows and columns of the array to shared data converters^15^; the recurrent connections require writing the outputs to a buffer memory outside of the RRAM array, and reading them back for the next time-step computation^35^.

**Fig. 2:**
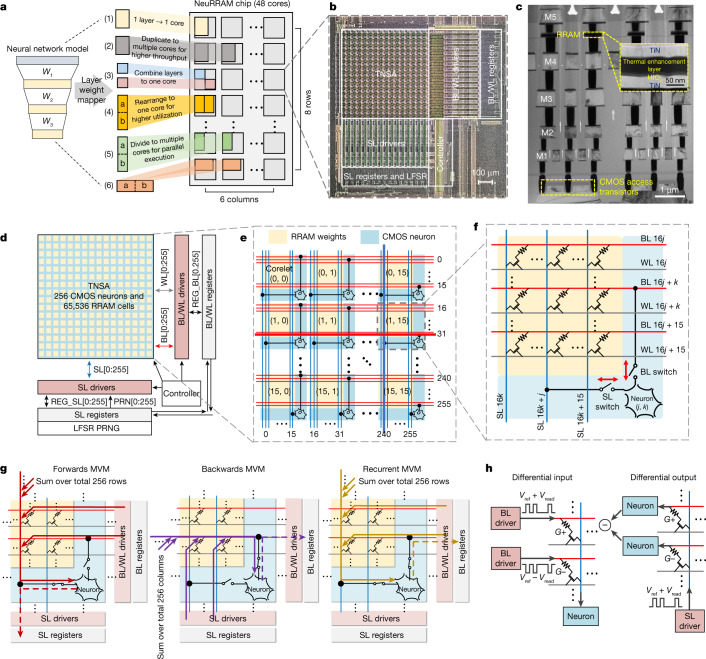
Reconfigurable architecture of the NeuRRAM chip. **a**, Multi-core architecture of the NeuRRAM chip, and various ways, labelled (1) to (6), to map neural-network layers onto CIM cores. **b**, Zoomed-in chip micrograph on a single CIM core. **c**, A cross-sectional transmission electron microscopy image showing the layer stack of the monolithically integrated RRAM and CMOS. **d**, Block diagram of a CIM core. A core consists of a TNSA, drivers for BLs, WLs, and SLs, registers that store MVM inputs and outputs, a LFSR pseudo-random number generator (PRNG), and a controller. During the MVM input stage, the drivers convert register inputs (REG) and PRNG inputs (PRN) to analogue voltages and send them to TNSA; during the MVM output stage, the drivers pass digital outputs from neurons back to registers through REG. **e**, The architecture of a TNSA consists of 16 × 16 corelets with interleaving RRAM weights and CMOS neurons. Each neuron integrates inputs from 256 RRAMs connecting to the same horizontal BL or vertical SL. **f**, Each corelet contains 16 × 16 RRAMs and 1 neuron. The neuron connects to 1 of the 16 BLs and 1 of the 16 SLs that pass through the corelet, and can use a BL and a SL for both its input and output. **g**, The TNSA can be dynamically configured for MVM in forwards, backwards or recurrent directions. **h**, Differential input and differential output schemes used to implement real-valued weights during forwards and backwards MVMs. Weights are encoded as the differential conductance between two RRAM cells on adjacent rows (G+ and G-).

The TNSA architecture realizes dynamic dataflow reconfigurability with little overhead. Whereas in conventional designs, CMOS peripheral circuits such as ADCs connect at only one end of the RRAM array, the TNSA architecture physically interleaves the RRAM weights and the CMOS neuron circuits, and connects them along the length of both rows and columns. As shown in Fig. 2e, a TNSA consists of 16 × 16 of such interleaved corelets that are connected by shared bit-lines (BLs) and word-lines (WLs) along the horizontal direction and source-lines (SLs) along the vertical direction. Each corelet encloses 16 × 16 RRAM devices and one neuron circuit. The neuron connects to 1 BL and 1 SL out of the 16 BLs and the 16 SLs that pass through the corelet, and is responsible for integrating inputs from all the 256 RRAMs connecting to the same BL or SL. Sixteen of these RRAMs are within the same corelet as the neuron; and the other 240 are within the other 15 corelets along the same row or column. Specifically, Fig. 2f shows that the neuron within corelet (*i*, *j*) connects to the (16*i* + *j*)th BL and the (16*j* *+* *i*)th SL. Such a configuration ensures that each BL or SL connects uniquely to a neuron, while doing so without duplicating neurons at both ends of the array, thus saving area and energy.

Moreover, a neuron uses its BL and SL switches for both its input and output: it not only receives the analogue MVM output coming from BL or SL through the switches but also sends the converted digital results to peripheral registers through the same switches. By configuring which switch to use during the input and output stages of the neuron, we can realize various MVM dataflow directions. Figure 2g shows the forwards, backwards and recurrent MVMs enabled by the TNSA. To implement forwards MVM (BL to SL), during the input stage, input pulses are applied to the BLs through the BL drivers, get weighted by the RRAMs and enter the neuron through its SL switch; during the output stage, the neuron sends the converted digital outputs to SL registers through its SL switch; to implement recurrent MVM (BL to BL), the neuron instead receives input through its SL switch and sends the digital output back to the BL registers through its BL switch.

Weights of most AI models take both positive and negative values. We encode each weight as difference of conductance between two RRAM cells on adjacent rows along the same column (Fig. 2h). The forwards MVM is performed using a differential input scheme, where BL drivers send input voltage pulses with opposite polarities to adjacent BLs. The backwards MVM is performed using a differential output scheme, where we digitally subtract outputs from neurons connecting to adjacent BLs after neurons finish analogue-to-digital conversions.

To maximize throughput of AI inference on 48 CIM cores, we implement a broad selection of weight-mapping strategies that allow us to exploit both model parallelism and data parallelism (Fig. 2a) through multi-core parallel MVMs. Using a CNN as an example, to maximize data parallelism, we duplicate the weights of the most computationally intensive layers (early convolutional layers) to multiple cores for parallel inference on multiple data; to maximize model parallelism, we map different convolutional layers to different cores and perform parallel inference in a pipelined fashion. Meanwhile, we divide the layers whose weight dimensions exceed the RRAM array size into multiple segments and assign them to multiple cores for parallel execution. A more detailed description of the weight-mapping strategies is provided in Methods. The intermediate data buffers and partial-sum accumulators are implemented by a field-programmable gate array (FPGA) integrated on the same board as the NeuRRAM chip. Although these digital peripheral modules are not the focus of this study, they will eventually need to be integrated within the same chip in production-ready RRAM-CIM hardware.

## Efficient voltage-mode neuron circuit

Figure 1d and Extended Data Table 1 show that the NeuRRAM chip achieves 1.6-times to 2.3-times lower EDP and 7-times to 13-times higher computational density (measured by throughput per million of RRAMs) at various MVM input and output bit-precisions than previous state-of-the-art RRAM-based CIM chips, despite being fabricated at an older technology node^17–27,36^. The reported energy and delay are measured for performing an MVM with a 256 × 256 weight matrix. It is noted that these numbers and those reported in previous RRAM-CIM work represent the peak energy efficiency achieved when the array utilization is 100% and does not account for energy spent on intermediate data transfer. Network-on-chip and program scheduling need to be carefully designed to achieve good end-to-end application-level energy efficiency^37,38^.

**Fig. 3:**
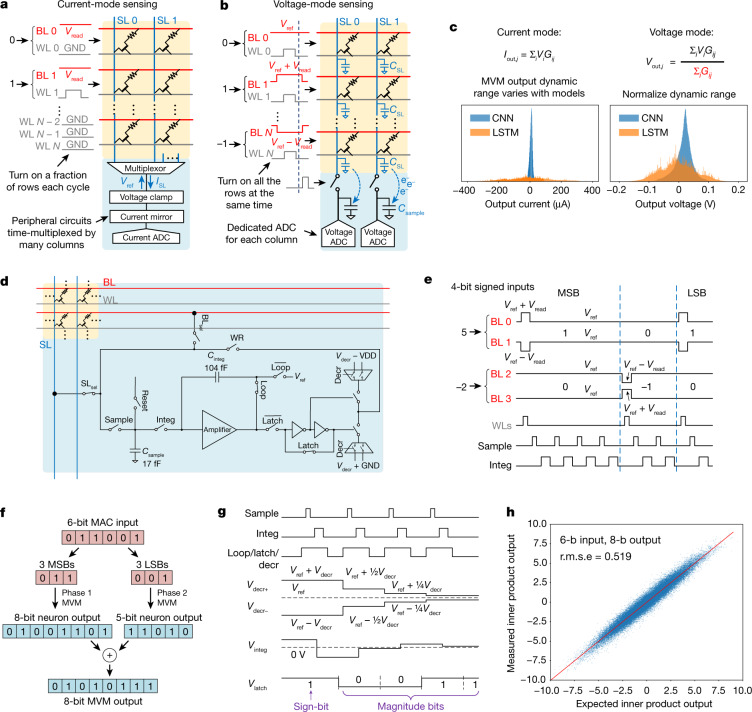
Voltage-mode MVM with multi-bit inputs and outputs. **a**, Conventional current-mode-sensing scheme needs to activate a small fraction of total *N* rows each cycle to limit total current *I*_SL _and time-multiplex ADCs across multiple columns to amortize ADC area, thus limiting its computational parallelism. **b**, Voltage-mode sensing employed by NeuRRAM can activate all the rows and all the columns in a single cycle, enabling higher parallelism. **c**, MVM output distribution from a CNN layer and from an LSTM layer (weights normalized to the same range). Voltage-mode sensing intrinsically normalizes wide variation in output dynamic range. **d**, Schematic of the voltage-mode neuron circuit, where BL_sel_, SL_sel_, Sample, Integ, Reset, Latch, Decr, and WR are digital signals controlling state of the switches. **e**, Sample waveforms to perform MVM and 4-bit signed inputs digital-to-analogue conversion. WLs are pulsed once per magnitude-bit; sampling and integration are performed 2^*n*−1^ times for the *n*th LSB. **f**, Two-phase MVM: for input precision greater than 4 bits, inputs are divided into a MSB segment and a LSB segment. MVMs and ADCs are performed separately for each segment, followed by a shift-and-add to obtain final outputs. **g**, Sample waveforms to perform 5-bit signed outputs analogue-to-digital conversion. The sign-bit is first generated by a comparison operation. The magnitude-bits are generated through a binary search process realized by adding/subtracting charge on *C*_integ_. From MSB to LSB, added/subtracted charge is halved every bit. **h**, Chip-measured 64 × 64 MVM outputs versus ideal outputs under 4-bit input and 6-bit output.

Key to the NeuRRAM’s EDP improvement is a novel in-memory MVM output-sensing scheme. The conventional approach is to use voltage as input, and measure the current as the results based on Ohm’s law (Fig. 3a). Such a current-mode-sensing scheme cannot fully exploit the high-parallelism nature of CIM. First, simultaneously turning on multiple rows leads to a large array current. Sinking the large current requires peripheral circuits to use large transistors, whose area needs to be amortized by time-multiplexing between multiple columns, which limits ‘column parallelism’. Second, MVM results produced by different neural-network layers have drastically different dynamic ranges (Fig. 3c). Optimizing ADCs across such a wide dynamic range is difficult. To equalize the dynamic range, designs typically activate a fraction of input wires every cycle to compute a partial sum, and thus require multiple cycles to complete an MVM, which limits ‘row parallelism’.

NeuRRAM improves computation parallelism and energy efficiency by virtue of a neuron circuit implementing a voltage-mode sensing scheme. The neuron performs analogue-to-digital conversion of the MVM outputs by directly sensing the settled open-circuit voltage on the BL or SL line capacitance^39^ (Fig. 3b): voltage inputs are driven on the BLs whereas the SLs are kept floating, or vice versa, depending on the MVM direction. WLs are activated to start the MVM operation. The voltage on the output line settles to the weighted average of the voltages driven on the input lines, where the weights are the RRAM conductances. Upon deactivating the WLs, the output is sampled by transferring the charge on the output line to the neuron sampling capacitor (*C*_sample_ in Fig. 3d). The neuron then accumulates this charge onto an integration capacitor (*C*_integ_) for subsequent analogue-to-digital conversion.

Such voltage-mode sensing obviates the need for power-hungry and area-hungry peripheral circuits to sink large current while clamping voltage, improving energy and area efficiency and eliminating output time-multiplexing. Meanwhile, the weight normalization owing to the conductance weighting in the voltage output (Fig. 3c) results in an automatic output dynamic range normalization for different weight matrices. Therefore, MVMs with different weight dimensions can all be completed within a single cycle, which significantly improves computational throughput. To eliminate the normalization factor from the final results, we pre-compute its value and multiply it back to the digital outputs from the ADC.

Our voltage-mode neuron supports MVM with 1-bit to 8-bit inputs and 1-bit to 10-bit outputs. The multi-bit input is realized in a bit-serial fashion where charge is sampled and integrated onto *C*_integ_ for 2^*n*−1^ cycles for the *n*th least significant bit (LSB) (Fig. 3e). For MVM inputs greater than 4 bits, we break the bit sequence into two segments, compute MVM for each segment separately and digitally perform a shift-and-add to obtain the final results (Fig. 3f). Such a two-phase input scheme improves energy efficiency and overcomes voltage headroom clipping at high-input precisions.

The multi-bit output is generated through a binary search process (Fig. 3g). Every cycle, neurons add or subtract *C*_sample_*V*_decr_ amount of charge from *C*_integ_, where *V*_decr_ is a bias voltage shared by all neurons. Neurons then compare the total charge on *C*_integ_ with a fixed threshold voltage *V*_ref_ to generate a 1-bit output. From the most significant bit (MSB) to the least significant bit (LSB), *V*_decr_ is halved every cycle. Compared with other ADC architectures that implement a binary search, our ADC scheme eliminates the residue amplifier of an algorithmic ADC, and does not require an individual DAC for each ADC to generate reference voltages like a successive approximation register (SAR) ADC^40^. Instead, our ADC scheme allows sharing a single digital-to-analogue converter (DAC) across all neurons to amortize the DAC area, leading to a more compact design. The multi-bit MVM is validated by comparing ideal and measured results, as shown in Fig. 3h and Extended Data Fig. 5. More details on the multi-bit input and output implementation can be found in Methods.

The neuron can also be reconfigured to directly implement Rectified Linear Unit (ReLU)/sigmoid/tanh as activations when needed. In addition, it supports probabilistic sampling for stochastic activation functions by injecting pseudo-random noise generated by a linear-feedback shift register (LFSR) block into the neuron integrator. All the neuron circuit operations are performed by dynamically configuring a single amplifier in the neuron as either an integrator or a comparator during different phases of operations, as detailed in Methods. This results in a more compact design than other work that merges ADC and neuron activation functions within the same module^12,13^. Although most existing CIM designs use time-multiplexed ADCs for multiple rows and columns to amortize the ADC area, the compactness of our neuron circuit allows us to dedicate a neuron for each pair of BL and SL, and tightly interleave the neuron with RRAM devices within the TNSA architecture, as can be seen in Extended Data Fig. 11d.

## Hardware-algorithm co-optimizations

**Fig. 4:**
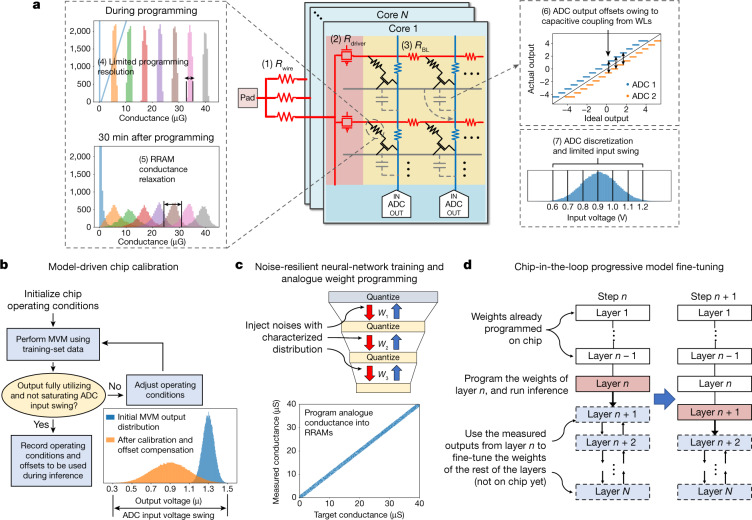
Hardware-algorithm co-optimization techniques to improve NeuRRAM inference accuracy. **a**, Various device and circuit non-idealities (labelled (1) to (7)) of in-memory MVM. **b**, Model-driven chip calibration technique to search for optimal chip operating conditions and record offsets for subsequent cancellation. **c**, Noise-resilient neural-network training technique to train the model with noise injection. The noise distribution is obtained from hardware characterization. The trained weights are programmed to the continuous analogue conductance of RRAMs without quantization as shown by the continuous diagonal band at the bottom. **d**, Chip-in-the-loop progressive fine-tuning technique: weights are progressively mapped onto the chip one layer at a time. The hardware-measured outputs from layer *n* are used as inputs to fine-tune the remaining layers *n* + 1 to *N*.

**Fig. 5:**
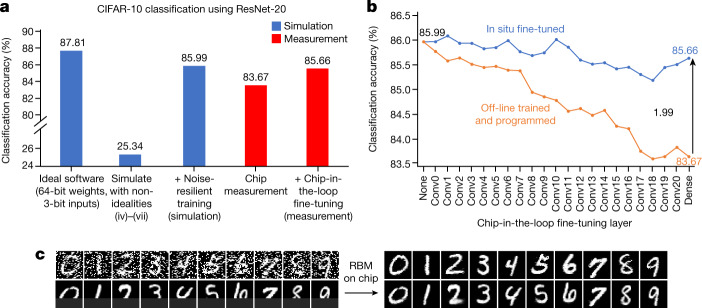
Measured results showing the efficacy of the hardware-algorithm co-optimization techniques. **a**, Simulated (blue) and measured (red) CIFAR-10 test-set classification accuracies. **b**, CIFAR-10 classification accuracy at various time steps of chip-in-the-loop fine-tuning. From left to right, each data point represents a new layer (Conv0 to Dense) programmed onto the chip. The accuracy at a layer is evaluated by using the hardware-measured outputs from that layer as inputs to the remaining layers that are simulated in software. Two curves compare the test-set inference accuracy with and without applying fine-tuning during training. **c**, RBM-based image recovery on noisy images (top) and partially occluded images measured on NeuRRAM (bottom).

The innovations on the chip architecture and circuit design bring superior efficiency and reconfigurability to NeuRRAM. To complete the story, we must ensure that AI inference accuracy can be preserved under various circuit and device non-idealities^3,41^. We developed a set of hardware-algorithm co-optimization techniques that allow NeuRRAM to deliver software-comparable accuracy across diverse AI applications. Importantly, all the AI benchmark results presented in this paper are obtained entirely from hardware measurements on complete datasets. Although most previous efforts (with a few exceptions^8,17^) have reported benchmark results using a mixture of hardware characterization and software simulation, for example, emulate the array-level MVM process in software using measured device characteristics^3,5,21,24^, such an approach often fails to model the complete set of non-idealities existing in realistic hardware. As shown in Fig. 4a, these non-idealities may include (1) Voltage drop on input wires (*R*_wire_), (2) on RRAM array drivers (*R*_driver_) and (3) on crossbar wires (e.g. BL resistance *R*_BL_), (4) limited RRAM programming resolution, (5) RRAM conductance relaxation^41^, (6) capacitive coupling from simultaneously switching array wires, and (7) limited ADC resolution and dynamic range. Our experiments show that omitting certain non-idealities in simulation leads to over-optimistic prediction of inference accuracy. For example, the third and the fourth bars in Fig. 5a show a 2.32% accuracy difference between simulation and measurement for CIFAR-10 classification^19^, whereas the simulation accounts for only non-idealities (5) and (7), which are what previous studies most often modelled^5,21^.

Our hardware-algorithm co-optimization approach includes three main techniques: (1) model-driven chip calibration, (2) noise-resilient neural-network training and analogue weight programming, and (3) chip-in-the-loop progressive model fine-tuning. Model-driven chip calibration uses the real model weights and input data to optimize chip operating conditions such as input voltage pulse amplitude, and records any ADC offsets for subsequent cancellation during inference. Ideally, the MVM output voltage dynamic range should fully utilize the ADC input swing to minimize discretization error. However, without calibration, the MVM output dynamic range varies with network layers even with the weight normalization effect of the voltage-mode sensing. To calibrate MVM to the optimal dynamic range, for each network layer, we use a subset of training-set data as calibration input to search for the best operating conditions (Fig. 4b). Extended Data Fig. 6 shows that different calibration input distributions lead to different output distributions. To ensure that the calibration data can closely emulate the distribution seen at test time, it is therefore crucial to use training-set data as opposed to randomly generated data during calibration. It is noted that when performing MVM on multiple cores in parallel, those shared bias voltages cannot be optimized for each core separately, which might lead to sub-optimal operating conditions and additional accuracy loss (detailed in Methods).

Stochastic non-idealities such as RRAM conductance relaxation and read noises degrade the signal-to-noise ratio (SNR) of the computation, leading to an inference accuracy drop. Some previous work obtained a higher SNR by limiting each RRAM cell to store a single bit, and encoding higher-precision weights using multiple cells^9,10,16^. Such an approach lowers the weight memory density. Accompanying that approach, the neural network is trained with weights quantized to the corresponding precision. In contrast, we utilize the intrinsic analogue programmability of RRAM^42^ to directly store high-precision weights and train the neural networks to tolerate the lower SNR. Instead of training with quantized weights, which is equivalent to injecting uniform noise into weights, we train the model with high-precision weights while injecting noise with the distribution measured from RRAM devices. RRAMs on NeuRRAM are characterized to have a Gaussian-distributed conductance spread, caused primarily by conductance relaxation. Therefore, we inject a Gaussian noise into weights during training, similar to a previous study^21^. Figure 5a shows that the technique significantly improves the model’s immunity to noise, from a CIFAR-10 classification accuracy of 25.34% without noise injection to 85.99% with noise injection. After the training, we program the non-quantized weights to RRAM analogue conductances using an iterative write–verify technique, described in Methods. This technique enables NeuRRAM to achieve an inference accuracy equivalent to models trained with 4-bit weights across various applications, while encoding each weight using only two RRAM cells, which is two-times denser than previous studies that require one RRAM cell per bit.

By applying the above two techniques, we already can measure inference accuracy comparable to or better than software models with 4-bit weights on Google speech command recognition, MNIST image recovery and MNIST classification (Fig. 1e). For deeper neural networks, we found that the error caused by those non-idealities that have nonlinear effects on MVM outputs, such as voltage drops, can accumulate through layers, and become more difficult to mitigate. In addition, multi-core parallel MVM leads to large instantaneous current, further exacerbating non-idealities such as voltage drop on input wires ((1) in Fig. 4a). As a result, when performing multi-core parallel inference on a deep CNN, ResNet-20^43^, the measured accuracy on CIFAR-10 classification (83.67%) is still 3.36% lower than that of a 4-bit-weight software model (87.03%).

To bridge this accuracy gap, we introduce a chip-in-the-loop progressive fine-tuning technique. Chip-in-the-loop training mitigates the impact of non-idealities by measuring training error directly on the chip^44^. Previous work has shown that fine-tuning the final layers using the back-propagated gradients calculated from hardware-measured outputs helped improve accuracy^5^. We find this technique to be of limited effectiveness in countering those nonlinear non-idealities. Such a technique also requires re-programming RRAM devices, which consumes additional energy. Our chip-in-the-loop progressive fine-tuning overcomes nonlinear model errors by exploiting the intrinsic nonlinear universal approximation capacity of the deep neural network^45^, and furthermore eliminates the need for weight re-programming. Figure 4d illustrates the fine-tuning procedure. We progressively program the weights one layer at a time onto the chip. After programming a layer, we perform inference using the training-set data on the chip up to that layer, and use the measured outputs to fine-tune the remaining layers that are still training in software. In the next time step, we program and measure the next layer on the chip. We repeat this process until all the layers are programmed. During the process, the non-idealities of the programmed layers can be progressively compensated by the remaining layers through training. Figure 5b shows the efficacy of this progressive fine-tuning technique. From left to right, each data point represents a new layer programmed onto the chip. The accuracy at each layer is evaluated by using the chip-measured outputs from that layer as inputs to the remaining layers in software. The cumulative CIFAR-10 test-set inference accuracy is improved by 1.99% using this technique. Extended Data Fig. 8a further illustrates the extent to which fine-tuning recovers the training-set accuracy loss at each layer, demonstrating the effectiveness of the approach in bridging the accuracy gap between software and hardware measurements.

Using the techniques described above, we achieve inference accuracy comparable to software models trained with 4-bit weights across all the measured AI benchmark tasks. Figure 1e shows that we achieve a 0.98% error rate on MNIST handwritten digit recognition using a 7-layer CNN, a 14.34% error rate on CIFAR-10 object classification using ResNet-20, a 15.34% error rate on Google speech command recognition using a 4-cell LSTM, and a 70% reduction of L2 image-reconstruction error compared with the original noisy images on MNIST image recovery using an RBM. Some of these numbers are not yet to the accuracies achieved by full-precision digital implementations. The accuracy gap mainly comes from low-precision (≤4-bit) quantization of inputs and activations, especially on the most sensitive input and output layers^46^. For instance, Extended Data Fig. 8b presents an ablation study that shows that quantizing input images to 4-bit alone results in a 2.7% accuracy drop for CIFAR-10 classification. By contrast, the input layer only accounts for 1.08% of compute and 0.16% of weights of a ResNet-20 model. Therefore, they can be off-loaded to higher-precision digital compute units with little overheads. In addition, applying more advanced quantization techniques and optimizing training procedures such as data augmentation and regularization should further improve the accuracy for both quantized software models and hardware-measured results.

Table 1 summarizes the key features of each demonstrated model. Most of the essential neural-network layers and operations are implemented on the chip, including all the convolutional, fully connected and recurrent layers, neuron activation functions, batch normalization and the stochastic sampling process. Other operations such as average pooling and element-wise multiplications are implemented on an FPGA integrated on the same board as NeuRRAM (Extended Data Fig. 11a). Each of the models is implemented by allocating the weights to multiple cores on a single NeuRRAM chip. We developed a software toolchain to allow easy deployment of AI models on the chip^47^. The implementation details are described in Methods. Fundamentally, each of the selected benchmarks represents a general class of common edge AI tasks: visual recognition, speech processing and image de-noising. These results demonstrate the versatility of the TNSA architecture and the wide applicability of the hardware-algorithm co-optimization techniques.

**Table 1:** Summary of AI applications and models demonstrated on NeuRRAM

Application	Dataset	Model architecture	Dataflow type	Activation precision	Number of parameters	Number of RRAMs used	Number of cores used	Average core utilization (%)
Image classification	CIFAR-10	ResNet-20 (CNN)	Forward	3-bit unsigned, input image 4-bit unsigned	274,461	553,524	48	17.6
	MNIST	7-layer CNN	Forwards	3-bit unsigned	23,170	46,664	16	4.5
Voice recognition	Google voice command	4 parallel LSTM cells	Recurrent + forwards	4-bit signed	281,392	570,048	36	24.2
Image recovery	MNIST	RBM	Forwards + backwards	Visible: 3-bit unsigned. Hidden: binary	96,194	200,880	8	38.3

The NeuRRAM chip simultaneously improves efficiency, flexibility and accuracy over existing RRAM-CIM hardware by innovating across the entire hierarchy of the design, from a TNSA architecture enabling reconfigurable dataflow direction, to an energy- and area-efficient voltage-mode neuron circuit, and to a series of algorithm-hardware co-optimization techniques. These techniques can be more generally applied to other non-volatile resistive memory technologies such as phase-change memory^8,17,21,23,24^, magnetoresistive RAM^48^ and ferroelectric field-effect transistors^49^. Going forwards, we expect NeuRRAM’s peak energy efficiency (EDP) to improve by another two to three orders of magnitude while supporting bigger AI models when scaling from 130-nm to 7-nm CMOS and RRAM technologies (detailed in Methods). Multi-core architecture design with network-on-chip that realizes efficient and versatile data transfers and inter-array pipelining is likely to be the next major challenge for RRAM-CIM^37,38^, which needs to be addressed by further cross-layer co-optimization. As resistive memory continues to scale towards offering tera-bits of on-chip memory^50^, such a co-optimization approach will equip CIM hardware on the edge with sufficient performance, efficiency and versatility to perform complex AI tasks that can only be done on the cloud today.

## Methods

### Core block diagram and operating modes

**Extended Data Fig. 1:**
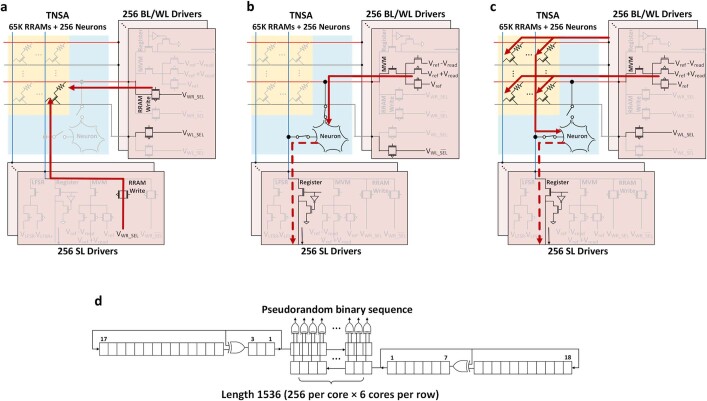
Peripheral driver circuits for TNSA and chip operating modes. **a**, driver circuits’ configuration under the weight-programming mode. **b**, under the neuron-testing mode. **c**, under the MVM mode. **d**, circuit diagram of the two counter-propagating LFSR chains XORed to generate pseudo-random sequences for probabilistic sampling.

**Extended Data Fig. 2:**
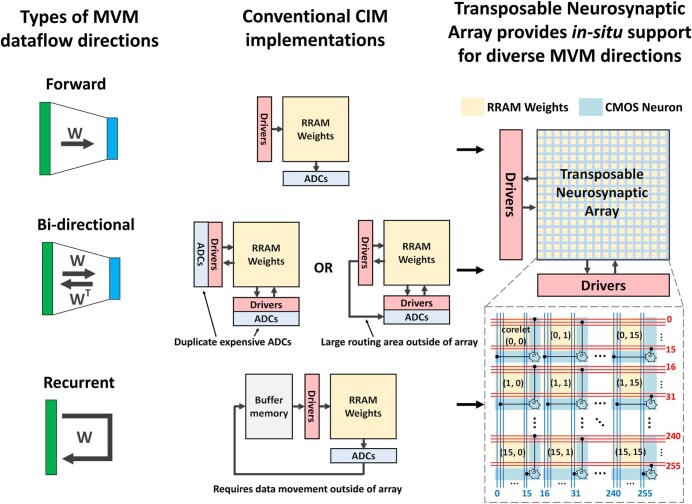
Various MVM dataflow directions and their CIM implementations. Left, various MVM dataflow directions commonly seen in different AI models. Middle, conventional CIM implementation of various dataflow directions. Conventional designs typically locate all peripheral circuits such as ADCs outside of RRAM array. The resulting implementations of bidirectional and recurrent MVMs incur overheads in area, latency, and energy. Right, the Transposable Neurosynaptic Array (TNSA) interleaves RRAM weights and CMOS neurons across the array and supports diverse MVM directions with minimal overhead.

Figure 2d and Extended Data Fig. 1 show the block diagram of a single CIM core. To support versatile MVM directions, most of the design is symmetrical in the row (BLs and WLs) and column (SLs) directions. The row and column register files store the inputs and outputs of MVMs, and can be written externally by either an Serial Peripheral Interface (SPI) or a random-access interface that uses an 8-bit address decoder to select one register entry, or internally by the neurons. The SL peripheral circuits contain an LFSR block used to generate pseudo-random sequences used for probabilistic sampling. It is implemented by two LFSR chains propagating in opposite directions. The registers of the two chains are XORed to generate spatially uncorrelated random numbers^51^. The controller block receives commands and generates control waveforms to the BL/WL/SL peripheral logic and to the neurons. It contains a delay-line-based pulse generator with tunable pulse width from 1 ns to 10 ns. It also implements clock-gating and power-gating logic used to turn off the core in idle mode. Each WL, BL and SL of the TNSA is driven by a driver consisting of multiple pass gates that supply different voltages. On the basis of the values stored in the register files and the control signals issued by the controller, the WL/BL/SL logic decides the state of each pass gate.

The core has three main operating modes: a weight-programming mode, a neuron-testing mode and an MVM mode (Extended Data Fig. 1). In the weight-programming mode, individual RRAM cells are selected for read and write. To select a single cell, the registers at the corresponding row and column are programmed to ‘1’ through random access with the help of the row and column decoder, whereas the other registers are reset to ‘0’. The WL/BL/SL logic turns on the corresponding driver pass gates to apply a set/reset/read voltage on the selected cell. In the neuron-testing mode, the WLs are kept at ground voltage (GND). Neurons receive inputs directly from BL or SL drivers through their BL or SL switch, bypassing RRAM devices. This allows us to characterize the neurons independently from the RRAM array. In the MVM mode, each input BL and SL is driven to *V*_ref_ − *V*_read_, *V*_ref_ + *V*_read_ or *V*_ref_ depending on the registers’ value at that row or column. If the MVM is in the BL-to-SL direction, we activate the WLs that are within the input vector length while keeping the rest at GND; if the MVM is in the SL-to-BL direction, we activate all the WLs. After neurons finish analogue-to-digital conversion, the pass gates from BLs and SLs to the registers are turned on to allow neuron-state readout.

### Device fabrication

RRAM arrays in NeuRRAM are in a one-transistor–one-resistor (1T1R) configuration, where each RRAM device is stacked on top of and connects in series with a selector NMOS transistor that cuts off the sneak path and provides current compliance during RRAM programming and reading. The selector n-type metal-oxide-semiconductor (NMOS), CMOS peripheral circuits and the bottom four back-end-of-line interconnect metal layers are fabricated in a standard 130-nm foundry process. Owing to the higher voltage required for RRAM forming and programming, the selector NMOS and the peripheral circuits that directly interface with RRAM arrays use thick-oxide input/output (I/O) transistors rated for 5-V operation. All the other CMOS circuits in neurons, digital logic, registers and so on use core transistors rated for 1.8-V operations.

The RRAM device is sandwiched between metal-4 and metal-5 layers shown in Fig. 2c. After the foundry completes the fabrication of CMOS and the bottom four metal layers, we use a laboratory process to finish the fabrication of the RRAM devices and the metal-5 interconnect, and the top metal pad and passivation layers. The RRAM device stack consists of a titanium nitride (TiN) bottom-electrode layer, a hafnium oxide (HfO_*x*_) switching layer, a tantalum oxide (TaO_*x*_) thermal-enhancement layer^52^ and a TiN top-electrode layer. They are deposited sequentially, followed by a lithography step to pattern the lateral structure of the device array.

### RRAM write–verify programming and conductance relaxation

Each neural-network weight is encoded by the differential conductance between two RRAM cells on adjacent rows along the same column. The first RRAM cell encodes positive weight, and is programmed to a low conductance state (*g*_min_) if the weight is negative; the second cell encodes negative weight, and is programmed to *g*_min_ if the weight is positive. Mathematically, the conductances of the two cells are ${\rm{\max }}({g}_{{\rm{\max }}}\frac{W}{{w}_{{\rm{\max }}}},{g}_{{\rm{\min }}})$ and ${\rm{\max }}(-{g}_{{\rm{\max }}}\frac{W}{{w}_{{\rm{\max }}}},{g}_{{\rm{\min }}})$ respectively, where *g*_max_ and *g*_min_ are the maximum and minimum conductance of the RRAMs, *w*_max_ is the maximum absolute value of weights, and *W* is the unquantized high-precision weight.

**Extended Data Fig. 3:**
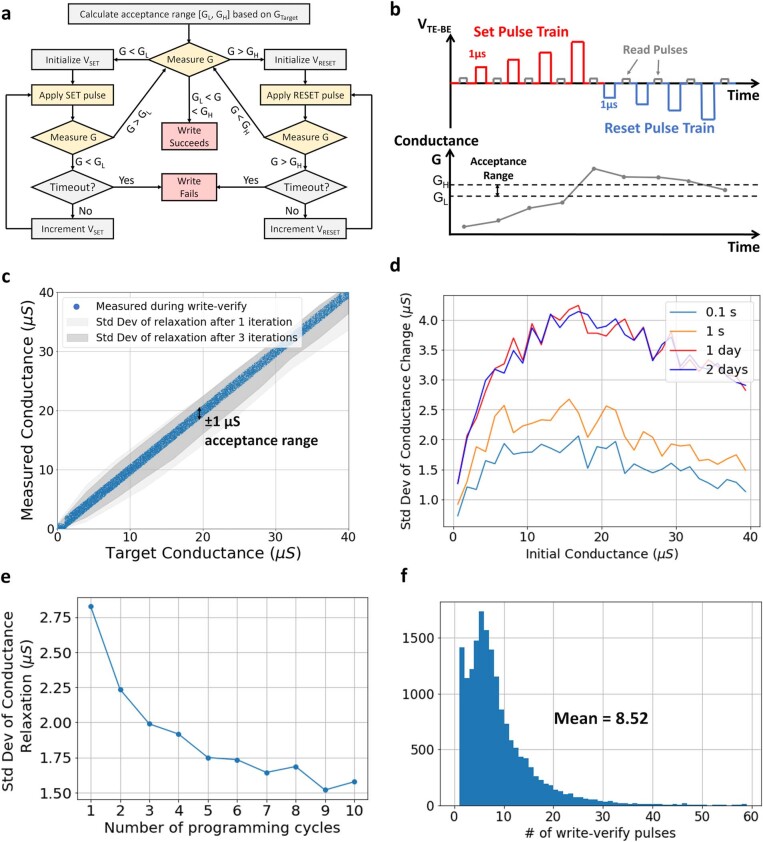
Iterative write–verify RRAM programming. **a**, Flowchart of the incremental-pulse write–verify technique to program RRAMs into target analogue conductance range. **b**, An example sequence of the write–verify programming. **c**, RRAM conductance distribution measured during and after the write–verify programming. Each blue dot represents one RRAM cell measured during write–verify. The grey shades show that the RRAM conductance relaxation cause the distribution to spread out from the target values. The darker shade shows that the iterative programming helps narrow the distribution. **d**, Standard deviation of conductance change measured at different initial conductance states and different time duration after the initial programming. The initial conductance relaxation happens at a faster rate than longer term retention degradation. **e**, Standard deviation of conductance relaxation decreases with increasing iterative programming cycles. **f**, Distribution of the number of SET/RESET pulses needed to reach conductance acceptance range.

To program an RRAM cell to its target conductance, we use an incremental-pulse write–verify technique^42^. Extended Data Fig. 3a,b illustrates the procedure. We start by measuring the initial conductance of the cell. If the value is below the target conductance, we apply a weak set pulse aiming to slightly increase the cell conductance. Then we read the cell again. If the value is still below the target, we apply another set pulse with amplitude incremented by a small amount. We repeat such set–read cycles until the cell conductance is within an acceptance range to the target value or overshoots to the other side of the target. In the latter case, we reverse the pulse polarity to reset, and repeat the same procedure as with set. During the set/reset pulse train, the cell conductance is likely to bounce up and down multiple times until eventually it enters the acceptance range or reaches a time-out limit.

There are a few trade-offs in selecting programming conditions. (1) A smaller acceptance range and a higher time-out limit improve programming precision, but require a longer time. (2) A higher *g*_max_ improves the SNR during inference, but leads to higher energy consumption and more programming failures for cells that cannot reach high conductance. In our experiments, we set the initial set pulse voltage to be 1.2 V and the reset pulse voltage to be 1.5 V, both with an increment of 0.1 V and pulse width of 1 μs. A RRAM read takes 1–10 μs, depending on its conductance. The acceptance range is ±1 μS to the target conductance. The time-out limit is 30 set–reset polarity reversals. We used *g*_min_ = 1 μS for all the models, and *g*_max_ = 40 μS for CNNs and *g*_max_ = 30 μS for LSTMs and RBMs. With such settings, 99% of the RRAM cells can be programmed to the acceptance range within the time-out limit. On average each cell requires 8.52 set/reset pulses. In the current implementation, the speed of such a write–verify process is limited by external control of DAC and ADC. If integrating everything into a single chip, such write–verify will take on average 56 µs per cell. Having multiple copies of DAC and ADC to perform write–verify on multiple cells in parallel will further improve RRAM programming throughput, at the cost of more chip area.

Besides the longer programming time, another reason to not use an overly small write–verify acceptance range is RRAM conductance relaxation. RRAM conductance changes over time after programming. Most of the change happens within a short time window (less than 1 s) immediately following the programming, after which the change becomes much slower, as shown in Extended Data Fig. 3d. The abrupt initial change is called ‘conductance relaxation’ in the literature^41^. Its statistics follow a Gaussian distribution at all conductance states except when the conductance is close to *g*_min_. Extended Data Fig. 3c,d shows the conductance relaxation measured across the whole *g*_min_-to*-g*_max_ conductance range. We found that the loss of programming precision owing to conductance relaxation is much higher than that caused by the write–verify acceptance range. The average standard deviation across all levels of initial conductance is about 2.8 μS. The maximum standard deviation is about 4 μS, which is close to 10% of *g*_max_.

To mitigate the relaxation, we use an iterative programming technique. We iterate over the RRAM array for multiple times. In each iteration, we measure all the cells and re-program those whose conductance has drifted outside the acceptance range. Extended Data Fig. 3e shows that the standard deviation becomes smaller with more programming iterations. After 3 iterations, the standard deviation becomes about 2 μS, a 29% decrease compared with the initial value. We use 3 iterations in all our neural-network demonstrations and perform inference at least 30 min after the programming such that the measured inference accuracy would account for such conductance relaxation effects. By combining the iterative programming with our hardware-aware model training approach, the impact of relaxation can be largely mitigated.

### Implementation of MVM with multi-bit inputs and outputs

**Extended Data Fig. 4:**
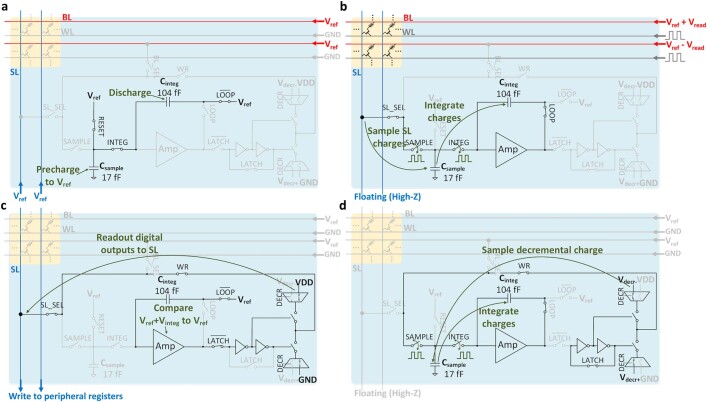
4 basic neuron operations that enable MVM with multi-bit inputs and outputs. **a**, Initialization, precharge sampling capacitor *C*_*sample*_ and output wires (SLs), and discharge integration capacitor *C*_*integ*_. **b**, Sampling and integration, sample SL voltage onto *C*_*sample*_, followed by integrating the charge onto *C*_*integ*_. **c**, Comparison and readout. The amplifier is turned into comparator mode to determine the polarity of the integrated voltage. Comparator outputs are written out of the neuron through the outer feedback loop. **d**, Charge decrement, charge is added or subtracted on *C*_*integ*_ through the outer feedback loop, depending on value stored in the latch.

The neuron and the peripheral circuits support MVM at configurable input and output bit-precisions. An MVM operation consists of an initialization phase, an input phase and an output phase. Extended Data Fig. 4 illustrates the neuron circuit operation. During the initialization phase (Extended Data Fig. 4a), all BLs and SLs are precharged to *V*_ref_. The sampling capacitors *C*_sample_ of the neurons are also precharged to *V*_ref_, whereas the integration capacitors *C*_integ_ are discharged.

During the input phase, each input wire (either BL or SL depending on MVM direction) is driven to one of three voltage levels, *V*_ref_ *−* *V*_read_, *V*_ref_ and *V*_ref_ *+* *V*_read_, through three pass gates, as shown in Fig. 3b. During forwards MVM, under differential-row weight mapping, each input is applied to a pair of adjacent BLs. The two BLs are driven to the opposite voltage with respect to *V*_ref_. That is, when the input is 0, both wires are driven to *V*_ref_; when the input is +1, the two wires are driven to *V*_ref_ + *V*_read_ and *V*_ref_ *−* *V*_read_; and when the input is *−*1, to *V*_ref_ *−* *V*_read_ and *V*_ref_ + *V*_read_. During backwards MVM, each input is applied to a single SL. The difference operation is performed digitally after neurons finish analogue-to-digital conversions.

After biasing the input wires, we then pulse those WLs that have inputs for 10 ns, while keeping output wires floating. As voltages of the output wires settle to ${V}_{j}=\frac{{\sum }_{i}{V}_{i}{G}_{{ij}}}{{\sum }_{i}{G}_{{ij}}}$, where *G*_ij_ represents conductance of RRAM at the *i-*th row and the *j-*th column, we turn off the WLs to stop all current flow. We then sample the charge remaining on the output wire parasitic capacitance to *C*_sample_ located within neurons, followed by integrating the charge onto *C*_integ_, as shown in Extended Data Fig. 4b. The sampling pulse is 10 ns (limited by the 100-MHz external clock from the FPGA); the integration pulse is 240 ns, limited by large integration capacitor (104 fF), which was chosen conservatively to ensure function correctness and testing different neuron operating conditions.

The multi-bit input digital-to-analogue conversion is performed in a bit-serial fashion. For the *n*th LSB, we apply a single pulse to the input wires, followed by sampling and integrating charge from output wires onto *C*_integ_ for 2^*n*−1^ cycles. At the end of multi-bit input phase, the complete analogue MVM output is stored as charge on *C*_integ_. For example, as shown in Fig. 3e, when the input vectors are 4-bit signed integers with 1 sign-bit and 3 magnitude-bits, we first send pulses corresponding to the first (least significant) magnitude-bit to input wires, followed by sampling and integrating for one cycle. For the second and the third magnitude-bits, we again apply one pulse to input wires for each bit, followed by sampling and integrating for two cycles and four cycles, respectively. In general, for *n*-bit signed integer inputs, we need a total of *n* − 1 input pulses and 2^*n*−1^ − 1 sampling and integration cycles.

**Extended Data Fig. 5:**
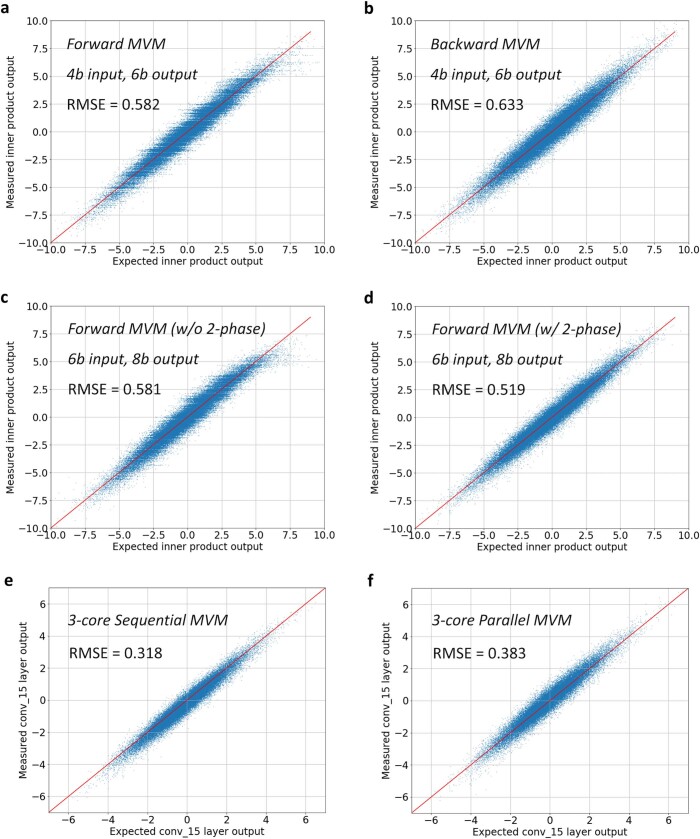
Scatter plots of measured MVMs vs. ideal MVMs. Results in a-d are generated using the same 64×64 normally distributed random matrix and 1000 uniformed distributed floating-point vectors ϵ [-1, 1]. **a**, Forward MVM using differential input scheme with inputs quantized to 4-bit and outputs 6-bit. **b**, Backward MVM using differential output scheme. The higher RMSE is caused by more voltage drop on each SL driver that needs to drive 128 RRAM cells, compared to 64 cells driven by each BL driver during forward MVM. **c**, MVM root-mean-square error (RMSE) does not reduce when increasing input from 4-bit (a) to 6-bit. This is caused by using a lower input voltage that leads to worse signal-to-noise-ratio. **d**, 2-phase operation reduces MVM RMSE with 6-bit input by breaking inputs into 2 segments and performing MVMs separately, such that input voltage does not need to be reduced. **e–f**, Outputs from conv15 layer of ResNet-20. Weights of conv15 are divided to 3 CIM cores. Layer outputs show a higher RMSE when performing MVM in parallel on the 3 cores (f) than sequentially on the 3 cores (e).

**Extended Data Fig. 6:**
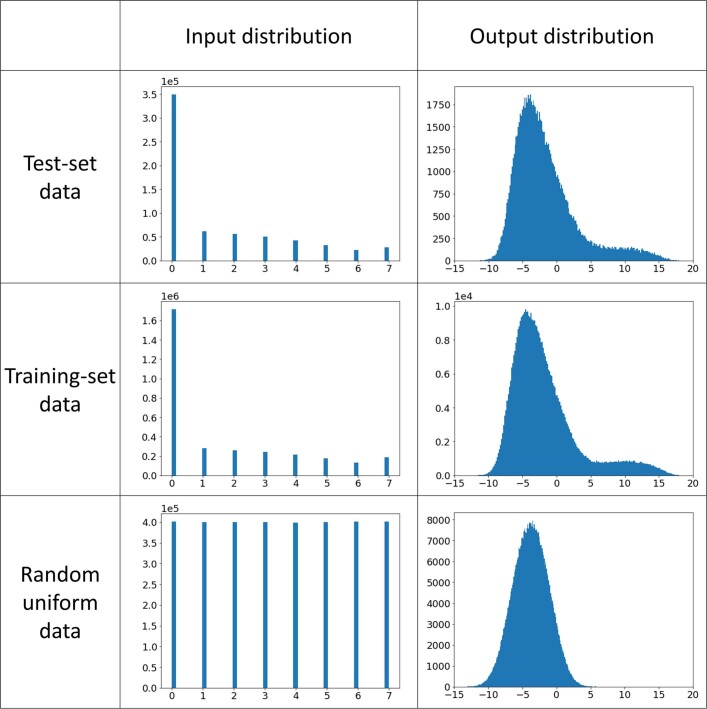
Data distribution with and without model-driven chip calibration. Left, Distribution of inputs to the final fully connected layer of ResNet-20 when the inputs are generated from (top-to-bottom) CIFAR-10 test-set data, training-set data, and random uniform data. Right, Distribution of outputs from the final fully connected layer of ResNet-20. The test-set and training-set have similar distributions while random uniform data produces a markedly different output distribution. To ensure that the MVM output voltage dynamic range during testing is calibrated to occupy the full ADC input swing, the calibration data should come from training-set data that closely resembles the test-set data.

Such a multi-bit input scheme becomes inefficient for high-input bit-precision owing to the exponentially increasing sampling and integration cycles. Moreover, headroom clipping becomes an issue as charge integrated at *C*_integ_ saturates with more integration cycles. The headroom clipping can be overcome by using lower *V*_read_, but at the cost of a lower SNR, so the overall MVM accuracy might not improve when using higher-precision inputs. For instance, Extended Data Fig. 5a,c shows the measured root-mean-square error (r.m.s.e.) of the MVM results. Quantizing inputs to 6-bit (r.m.s.e. = 0.581) does not improve the MVM accuracy compared with 4-bit (r.m.s.e. = 0.582), owing to the lower SNR.

To solve both the issues, we use a 2-phase input scheme for input greater than 4-bits. Figure 3f illustrates the process. To perform MVM with 6-bit inputs and 8-bit outputs, we divide inputs into two segments, the first containing the three MSBs and the second containing the three LSBs. We then perform MVM including the output analogue-to-digital conversion for each segment separately. For the MSBs, neurons (ADCs) are configured to output 8-bits; for the LSBs, neurons output 5-bits. The final results are obtained by shifting and adding the two outputs in digital domain. Extended Data Fig. 5d shows that the scheme lowers MVM r.m.s.e. from 0.581 to 0.519. Extended Data Fig. 12c–e further shows that such a two-phase scheme both extends the input bit-precision range and improves the energy efficiency.

Finally, during the output phase, the analogue-to-digital conversion is again performed in a bit-serial fashion through a binary search process. First, to generate the sign-bit of outputs, we disconnect the feedback loop of the amplifier to turn the integrator into a comparator (Extended Data Fig. 4c). We drive the right side of *C*_integ_ to *V*_ref_. If the integrated charge is positive, the comparator output will be GND, and supply voltage VDD otherwise. The comparator output is then inverted, latched and readout to the BL or SL via the neuron BL or SL switch before being written into the peripheral BL or SL registers.

To generate *k* magnitude-bits, we add or subtract charge from *C*_integ_ (Extended Data Fig. 4d), followed by comparison and readout for *k* cycles. From MSB to LSB, the amount of charge added or subtracted is halved every cycle. Whether to add or to subtract is automatically determined by the comparison result stored in the latch from the previous cycle. Figure 3g illustrates such a process. A sign-bit of ‘1’ is first generated and latched in the first cycle, representing a positive output. To generate the most significant magnitude-bit, the latch turns on the path from *V*_decr−_ = *V*_ref_ − *V*_decr_ to *C*_sample_. The charge sampled by *C*_sample_ is then integrated on *C*_integ_ by turning on the negative feedback loop of the amplifier, resulting in *C*_sample_*V*_decr_ amount of charge being subtracted from *C*_integ_. In this example, *C*_sample_*V*_decr_ is greater than the original amount of charge on *C*_integ_, so the total charge becomes negative, and the comparator generates a ‘0’ output. To generate the second magnitude-bit, *V*_decr_ is reduced by half. This time, the latch turns on the path from *V*_decr+_ = *V*_ref_ + 1/2*V*_decr_ to *C*_sample_. As the total charge on *C*_integ_ after integration is still negative, the comparator outputs a ‘0’ again in this cycle. We repeat this process until the least significant magnitude-bit is generated. It is noted that if the initial sign-bit is ‘0’, all subsequent magnitude-bits are inverted before readout.

Such an output conversion scheme is similar to an algorithmic ADC or a SAR ADC in the sense that a binary search is performed for *n* cycles for a *n*-bit output. The difference is that an algorithmic ADC uses a residue amplifier, and a SAR ADC requires a multi-bit DAC for each ADC, whereas our scheme does not need a residue amplifier, and uses a single DAC that outputs 2 × (*n* − 1) different *V*_decr+_ and *V*_decr−_ levels, shared by all neurons (ADCs). As a result, our scheme enables a more compact design by time-multiplexing an amplifier for integration and comparison, eliminating the residual amplifier, and amortizing the DAC area across all neurons in a CIM core. For CIM designs that use a dense memory array, such a compact design allows each ADC to be time-multiplexed by a fewer number of rows and columns, thus improving throughput.

To summarize, both the configurable MVM input and output bit-precisions and various neuron activation functions are implemented using different combinations of the four basic operations: sampling, integration, comparison and charge decrement. Importantly, all the four operations are realized by a single amplifier configured in different feedback modes. As a result, the design realizes versatility and compactness at the same time.

### Multi-core parallel MVM

NeuRRAM supports performing MVMs in parallel on multiple CIM cores. Multi-core MVM brings additional challenges to computational accuracy, because certain hardware non-idealities that do not manifest in single-core MVM become more severe with more cores. They include voltage drop on input wires, core-to-core variation and supply voltage instability. voltage drop on input wires (non-ideality (1) in Fig. 4a) is caused by large current drawn from a shared voltage source simultaneously by multiple cores. It makes equivalent weights stored in each core vary with applied inputs, and therefore have a nonlinear input-dependent effect on MVM outputs. Moreover, as different cores have a different distance from the shared voltage source, they experience a different amounts of voltage drops. Therefore, we cannot optimize read-voltage amplitude separately for each core to make its MVM output occupy exactly the full neuron input dynamic range.

These non-idealities together degrade the multi-core MVM accuracy. Extended Data Fig. 5e,f shows that when performing convolution in parallel on the 3 cores, outputs of convolutional layer 15 are measured to have a higher r.m.s.e. of 0.383 compared with 0.318 obtained by performing convolution sequentially on the 3 cores. In our ResNet-20 experiment, we performed 2-core parallel MVMs for convolutions within block 1 (Extended Data Fig. 9a), and 3-core parallel MVMs for convolutions within blocks 2 and 3.

The voltage-drop issue can be partially alleviated by making the wires that carry large instantaneous current as low resistance as possible, and by employing a power delivery network with more optimized topology. But the issue will persist and become worse as more cores are used. Therefore, our experiments aim to study the efficacy of algorithm-hardware co-optimization techniques in mitigating the issue. Also, it is noted that for a full-chip implementation, additional modules such as intermediate result buffers, partial-sum accumulators and network-on-chip will need to be integrated to manage inter-core data transfers. Program scheduling should also be carefully optimized to minimize buffer size and energy spent at intermediate data movement. Although there are studies on such full-chip architecture and scheduling^37,38,53^, they are outside the scope of this study.

### Noise-resilient neural-network training

During noise-resilient neural-network training, we inject noise into weights of all fully connected and convolutional layers during the forwards pass of neural-network training to emulate the effects of RRAM conductance relaxation and read noises. The distribution of the injected noise is obtained by RRAM characterization. We used the iterative write–verify technique to program RRAM cells into different initial conductance states and measure their conductance relaxation after 30 min. Extended Data Fig. 3d shows that measured conductance relaxation has an absolute value of mean <1 μS (*g*_min_) at all conductance states. The highest standard deviation is 3.87 μS, about 10% of the *g*_max_ 40 μS, found at about 12 μS initial conductance state. Therefore, to simulate such conductance relaxation behaviour during inference, we inject a Gaussian noise with a zero mean and a standard deviation equal to 10% of the maximum weights of a layer.

**Extended Data Fig. 7:**
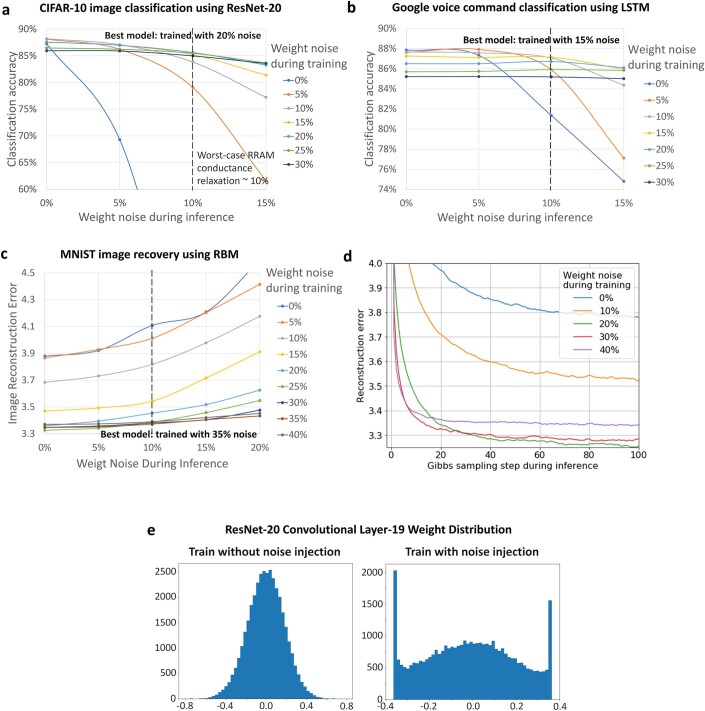
Noise-resilient training of CNNs, LSTMs and RBMs. **a**, Change in CIFAR-10 test-set classification accuracy under different weight noise levels during inference. Noise is represented as fraction of the maximum absolute value of weights. Different curves represent models trained at different levels of noise injection. **b**, Change in voice command recognition accuracy with weight noise levels. **c**, Change in MNIST image-reconstruction error with weight noise levels. **d**, Decreasing of image-reconstruction error with Gibbs sampling steps during RBM inference. **e**, Differences in weight distributions when trained without and with noise injection.

**Extended Data Fig. 8:**
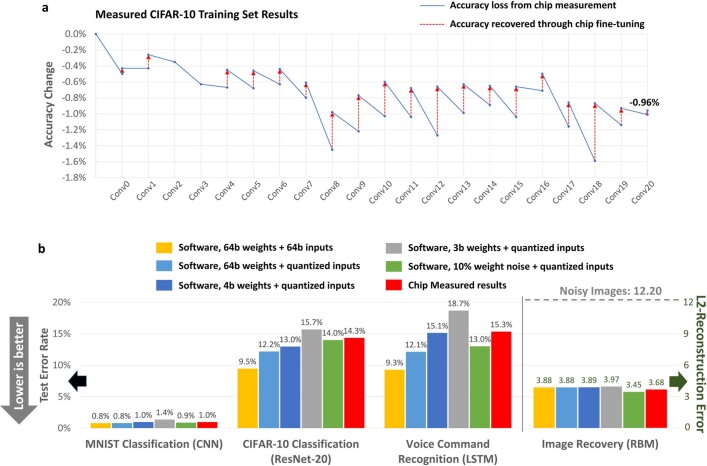
Measured chip inference performance. **a**, CIFAR-10 training-set accuracy loss due to hardware non-idealities, and accuracy recovery at each step of the chip-in-the-loop progressive fine-tuning. From left to right, each data point represents a new layer programmed onto the chip. The blue solid lines represent the accuracy loss measured when performing inference of that layer on-chip. The red dotted lines represent the measured recovery in accuracy by fine-tuning subsequent layers. **b**, Ablation study showing the impacts of input, activation, and weight quantizations, and weight noise injection on inference errors.

We train models with different levels of noise injection from 0% to 40%, and select the model that achieves the highest inference accuracy at 10% noise level for on-chip deployment. We find that injecting a higher noise during training than testing improves models’ noise resiliency. Extended Data Fig. 7a–c shows that the best test-time accuracy in the presence of 10% weight noise is obtained with 20% training-time noise injection for CIFAR-10 image classification, 15% for Google voice command classification and 35% for RBM-based image reconstruction.

For CIFAR-10, the better initial accuracy obtained by the model trained with 5% noise is most likely due to the regularization effect of noise injection. A similar phenomenon has been reported in neural-network quantization literature where a model trained with quantization occasionally outperforms a full-precision model^54,55^. In our experiments, we did not apply additional regularization on top of noise injection for models trained without noise, which might result in sub-optimal accuracy.

For RBM, Extended Data Fig. 7d further shows how reconstruction errors reduce with the number of Gibbs sampling steps for models trained with different noises. In general, models trained with higher noises converge faster during inference. The model trained with 20% noise reaches the lowest error at the end of 100 Gibbs sampling steps.

Extended Data Fig. 7e shows the effect of noise injection on weight distribution. Without noise injection, the weights have a Gaussian distribution. The neural-network outputs heavily depend on a small fraction of large weights, and thus become vulnerable to noise injection. With noise injection, the weights distribute more uniformly, making the model more noise resilient.

To efficiently implement the models on NeuRRAM, inputs to all convolutional and fully connected layers are quantized to 4-bit or below. The input bit-precisions of all the models are summarized in Table 1. We perform the quantized training using the parameterized clipping activation technique^46^. The accuracies of some of our quantized models are lower than that of the state-of-the-art quantized model because we apply <4-bit quantization to the most sensitive input and output layers of the neural networks, which have been reported to cause large accuracy degradation and are thus often excluded from low-precision quantization^46,54^. To obtain better accuracy for quantized models, one can use higher precision for sensitive input and output layers, apply more advanced quantization techniques, and use more optimized data preprocessing, data augmentation and regularization techniques during training. However, the focus of this work is to achieve comparable inference accuracy on hardware and on software while keeping all these variables the same, rather than to obtain state-of-the-art inference accuracy on all the tasks. The aforementioned quantization and training techniques will be equally beneficial for both our software baselines and hardware measurements.

### Chip-in-the-loop progressive fine-tuning

During the progressive chip-in-the-loop fine-tuning, we use the chip-measured intermediate outputs from a layer to fine-tune the weights of the remaining layers. Importantly, to fairly evaluate the efficacy of the technique, we do not use the test-set data (for either training or selecting checkpoint) during the entire process of fine-tuning. To avoid over-fitting to a small fraction of data, measurements should be performed on the entire training-set data. We reduce the learning rate to 1/100 of the initial learning rate used for training the baseline model, and fine-tune for 30 epochs, although we observed that the accuracy generally plateaus within the first 10 epochs. The same weight noise injection and input quantization are applied during the fine-tuning.

### Implementations of CNNs, LSTMs and RBMs

**Extended Data Fig. 9:**
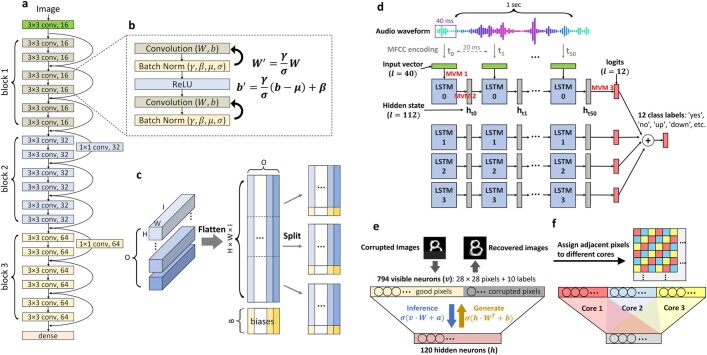
Implementation of various AI models. **a**, Architecture of ResNet-20 for CIFAR-10 classification. **b**, The batch normalization parameters are merged into convolutional weights and biases before mapping on-chip. **c**, Illustration of the process to map 4-dimensional weights of a convolutional layer to NeuRRAM CIM cores. **d**, Architecture of the LSTM model used for Google speech command recognition. The model contains 4 parallel LSTM cells and makes predictions based on the sum of outputs from the 4 cells. **e**, Architecture of the RBM model used for MNIST image recovery. During inference, MVMs and Gibbs sampling are performed back and-forth between visible and hidden neurons. **f**, Process to map RBM on NeuRRAM CIM cores. Adjacent pixels are assigned to different cores to equalize the MVM output dynamic range at different cores.

We use CNN models for the CIFAR-10 and MNIST image classification tasks. The CIFAR-10 dataset consists of 50,000 training images and 10,000 testing images belonging to 10 object classes. We perform image classification using the ResNet-20^43^, which contains 21 convolutional layers and 1 fully connected layer (Extended Data Fig. 9a), with batch normalizations and ReLU activations between the layers. The model is trained using the Keras framework. We quantize the input of all convolutional and fully connected layers to a 3-bit unsigned fixed-point format except for the first convolutional layer, where we quantize the input image to 4-bit because the inference accuracy is more sensitive to the input quantization. For the MNIST handwritten digits classification, we use a seven-layer CNN consisting of six convolutional layers and one fully connected layer, and use max-pooling between layers to down-sample feature map sizes. The inputs to all the layers, including the input image, are quantized to a 3-bit unsigned fixed-point format.

All the parameters of the CNNs are implemented on a single NeuRRAM chip including those of the convolutional layers, the fully connected layers and the batch normalization. Other operations such as partial-sum accumulation and average pooling are implemented on an FPGA integrated on the same board as the NeuRRAM. These operations amount to only a small fraction of the total computation and integrating their implementation in digital CMOS would incur negligible overhead; the FPGA implementation was chosen to provide greater flexibility during test and development.

Extended Data Fig. 9a–c illustrates the process to map a convolutional layer on a chip. To implement the weights of a four-dimensional convolutional layer with dimension *H* (height), *W* (width), *I* (number of input channels), *O* (number of output channels) on two-dimensional RRAM arrays, we flatten the first three dimensions into a one-dimensional vector, and append the bias term of each output channel to each vector. If the range of the bias values is *B* times of the weight range, we evenly divide the bias values and implement them using *B* rows. Furthermore, we merge the batch normalization parameters into convolutional weights and biases after training (Extended Data Fig. 9b), and program the merged *W*ʹ and *b*ʹ onto RRAM arrays such that no explicit batch normalization needs to be performed during inference.

Under the differential-row weight-mapping scheme, the parameters of a convolutional layer are converted into a conductance matrix of size (2(*HWI* + *B*), *O*). If the conductance matrix fits into a single core, an input vector is applied to 2(*HWI* + *B*) rows and broadcast to *O* columns in a single cycle. *HWIO* multiply–accumulate (MAC) operations are performed in parallel. Most ResNet-20 convolutional layers have a conductance matrix height of 2(*HWI* + *B*) that is greater than the RRAM array length of 256. We therefore split them vertically into multiple segments, and map the segments either onto different cores that are accessed in parallel, or onto different columns within a core that are accessed sequentially. The details of the weight-mapping strategies are described in the next section.

The Google speech command dataset consists of 65,000 1-s-long audio recordings of voice commands, such as ‘yes’, ‘up’, ‘on’, ‘stop’ and so on, spoken by thousands of different people. The commands are categorized into 12 classes. Extended Data Fig. 9d illustrates the model architecture. We use the Mel-frequency cepstral coefficient encoding approach to encode every 40-ms piece of audio into a length-40 vector. With a hop length of 20 ms, we have a time series of 50 steps for each 1-s recording.

We build a model that contains four parallel LSTM cells. Each cell has a hidden state of length 112. The final classification is based on summation of outputs from the four cells. Compared with a single-cell model, the 4-cell model reduces the classification error (of an unquantized model) from 10.13% to 9.28% by leveraging additional cores on the NeuRRAM chip. Within a cell, in each time step, we compute the values of four LSTM gates (input, activation, forget and output) based on the inputs from the current step and hidden states from the previous step. We then perform element-wise operations between the four gates to compute the new hidden-state value. The final logit outputs are calculated based on the hidden states of the final time step.

Each LSTM cell has 3 weight matrices that are implemented on the chip: an input-to-hidden-state matrix with size 40 × 448, a hidden-state-to-hidden-state matrix with size 112 × 448 and a hidden-state-to-logits matrix with size 112 × 12. The element-wise operations are implemented on the FPGA. The model is trained using the PyTorch framework. The inputs to all the MVMs are quantized to 4-bit signed fixed-point formats. All the remaining operations are quantized to 8-bit.

An RBM is a type of generative probabilistic graphical model. Instead of being trained to perform discriminative tasks such as classification, it learns the statistical structure of the data itself. Extended Data Fig. 9e shows the architecture of our image-recovery RBM. The model consists of 794 fully connected visible neurons, corresponding to 784 image pixels plus 10 one-hot encoded class labels and 120 hidden neurons. We train the RBM using the contrastive divergence learning procedure in software.

**Extended Data Fig. 10:**
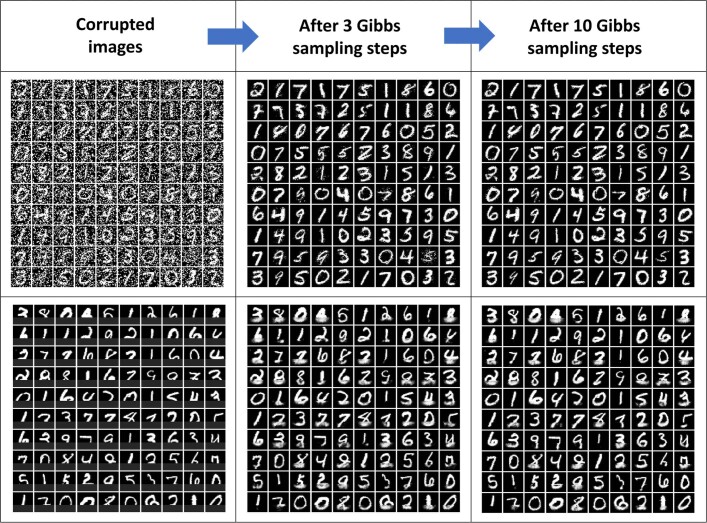
Chip-measured image recovery using RBM. Top: Recovery of MNIST test-set images with randomly selected 20% of pixels flipped to complementary intensity. Bottom: Recovery of MNIST test-set images with bottom 1/3 of pixels occluded.

During inference, we send 3-bit images with partially corrupted or blocked pixels to the model running on a NeuRRAM chip. The model then performs back-and-forth MVMs and Gibbs sampling between visible and hidden neurons for ten cycles. In each cycle, neurons sample binary states *h* and *v* from the MVM outputs based on the probability distributions: $p({h}_{j}=1| {\bf{v}})=\sigma ({b}_{j}+{\sum }_{i}{v}_{i}{w}_{ij})$ and $p({h}_{j}=1| {\bf{v}})=$ $\sigma ({b}_{j}+{\sum }_{i}{v}_{i}{w}_{ij})$, where *σ* is the sigmoid function, *a*_*i*_ is a bias for hidden neurons (*h*) and *b*_*j*_ is a bias for visible neurons (*v*). After sampling, we reset the uncorrupted pixels (visible neurons) to the original pixel values. The final inference performance is evaluated by computing the average L2-reconstruction error between the original image and the recovered image. Extended Data Fig. 10 shows some examples of the measured image recovery.

When mapping the 794 × 120 weight matrix to multiple cores of the chip, we try to make the MVM output dynamic range of each core relatively consistent such that the recovery performance will not overly rely on the computational accuracy of any single core. To achieve this, we assign adjacent pixels (visible neurons) to different cores such that every core sees a down-sampled version of the whole image, as shown in Extended Data Fig. 9f). Utilizing the bidirectional MVM functionality of the TNSA, the visible-to-hidden neuron MVM is performed from the SL-to-BL direction in each core; the hidden-to-visible neuron MVM is performed from the BL-to-SL direction.

### Weight-mapping strategy onto multiple CIM cores

To implement an AI model on a NeuRRAM chip, we convert the weights, biases and other relevant parameters (for example, batch normalization) of each model layer into a single two-dimensional conductance matrix as described in the previous section. If the height or the width of a matrix exceed the RRAM array size of a single CIM core (256 × 256), we split the matrix into multiple smaller conductance matrices, each with maximum height and width of 256.

We consider three factors when mapping these conductance matrices onto the 48 cores: resource utilization, computational load balancing and voltage drop. The top priority is to ensure that all conductance matrices of a model are mapped onto a single chip such that no re-programming is needed during inference. If the total number of conductance matrices does not exceed 48, we can map each matrix onto a single core (case (1) in Fig. 2a) or multiple cores. There are two scenarios when we map a single matrix onto multiple cores. (1) When a model has different computational intensities, defined as the amount of computation per weights, for different layers, for example, CNNs often have higher computational intensity for earlier layers owing to larger feature map dimensions, we duplicate the more computationally intensive matrices to multiple cores and operate them in parallel to increase throughput and balance the computational loads across the layers (case (2) in Fig. 2a). (2) Some models have ‘wide’ conductance matrices (output dimension >128), such as our image-recovery RBM. If mapping the entire matrix onto a single core, each input driver needs to supply large current for its connecting RRAMs, resulting in a significant voltage drop on the driver, deteriorating inference accuracy. Therefore, when there are spare cores, we can split the matrix vertically into multiple segments and map them onto different cores to mitigate the voltage drop (case (6) in Fig. 2a).

By contrast, if a model has more than 48 conductance matrices, we need to merge some matrices so that they can fit onto a single chip. The smaller matrices are merged diagonally such that they can be accessed in parallel (case (3) in Fig. 2a). The bigger matrices are merged horizontally and accessed by time-multiplexing input rows (case (4) in Fig. 2a). When selecting the matrices to merge, we want to avoid the matrices that belong to the same two categories described in the previous paragraph: (1) those that have high computational intensity (for example, early layers of ResNet-20) to minimize impact on throughput; and (2) those with ‘wide’ output dimension (for example, late layers of ResNet-20 have large number of output channels) to avoid a large voltage drop. For instance, in our ResNet-20 implementation, among a total of 61 conductance matrices (Extended Data Fig. 9a: 1 from input layer, 12 from block 1, 17 from block 2, 28 from block 3, 2 from shortcut layers and 1 from final dense layer), we map each of the conductance matrices in blocks 1 and 3 onto a single core, and merge the remaining matrices to occupy the 8 remaining cores.

Table 1 summarizes core usage for all the models. It is noted that for partially occupied cores, unused RRAM cells are either unformed or programmed to high resistance state; WLs of unused rows are not activated during inference. Therefore, they do not consume additional energy during inference.

### Test-system implementation

**Extended Data Fig. 11:**
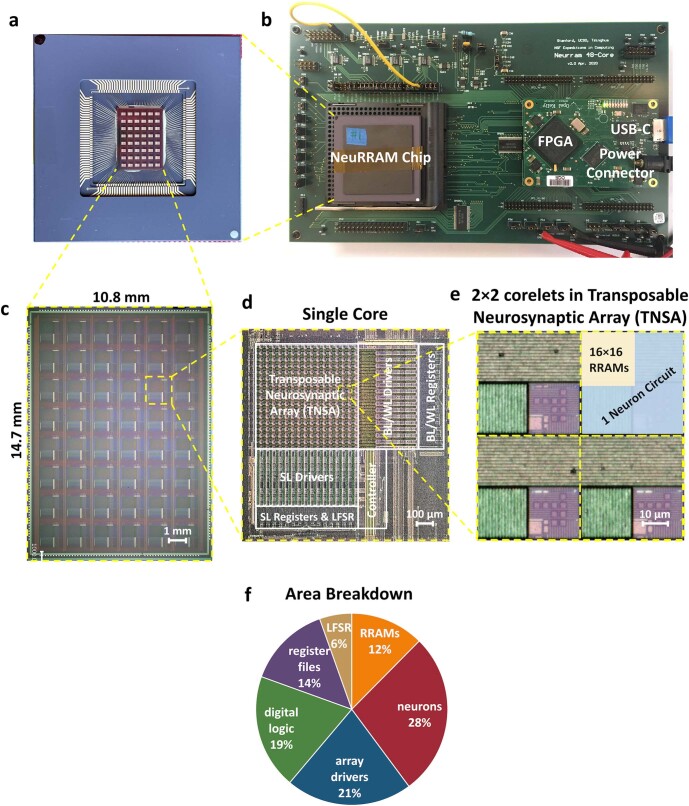
NeuRRAM test system and chip micrographs at various scales. **a**, A NeuRRAM chip wire-bonded to a package. **b**, Measurement board that connects a packaged NeuRRAM chip (left) to a field-programmable gate array (FPGA, right). The board houses all the components necessary to power, operate and measure the chip. No external lab equipment is needed for the chip operations. **c**, Micrograph of a 48-core NeuRRAM chip. **d**, Zoomed-in micrograph of a single CIM core. **e**, Zoomed-in micrograph of 2×2 corelets within the TNSA. One neuron circuit occupies 1270 μm^2^, which is >100× smaller than most ADC designs in 130-nm summarized in an ADC survey^62^. **f**, Chip area breakdown.

Extended Data Fig. 11a shows the hardware test system for the NeuRRAM chip. The NeuRRAM chip is configured by, receives inputs from and sends outputs to a Xilinx Spartan-6 FPGA that sits on an Opal Kelly integrated FPGA board. The FPGA communicates with the PC via a USB 3.0 module. The test board also houses voltage DACs that provide various bias voltages required by RRAM programming and MVM, and ADCs to measure RRAM conductance during the write–verify programming. The power of the entire board is supplied by a standard ‘cannon style’ d.c. power connector and integrated switching regulators on the Opal Kelly board such that no external lab equipment is needed for the chip operation.

To enable fast implementation of various machine-learning applications on the NeuRRAM chip, we developed a software toolchain that provides Python-based application programming interfaces (APIs) at various levels. The low-level APIs provide access to basic operations of each chip module such as RRAM read and write and neuron analogue-to-digital conversion; the middle-level APIs include essential operations required for implementing neural-network layers such as the multi-core parallel MVMs with configurable bit-precision and RRAM write–verify programming; the high-level APIs integrate various middle-level modules to provide complete implementations of neural-network layers, such as weight mapping and batch inference of convolutional and fully connected layers. The software toolchain aims to allow software developers who are not familiar with the NeuRRAM chip design to deploy their machine-learning models on the NeuRRAM chip.

### Power and throughput measurements

To characterize MVM energy efficiency at various input and output bit-precisions, we measure the power consumption and latency of the MVM input and output stages separately. The total energy consumption and the total time are the sum of input and output stages because the two stages are performed independently as described in the above sections. As a result, we can easily obtain the energy efficiency of any combinations of input and output bit-precisions.

To measure the input-stage energy efficiency, we generate a 256 × 256 random weight matrix with Gaussian distribution, split it into 2 segments, each with dimension 128 × 256, and program the two segments to two cores using the differential-row weight mapping. We measure the power consumption and latency for performing 10 million MVMs, or equivalently 655 billion MAC operations. The comparison with previous work shown in Fig. 1d uses the same workload as benchmark.

**Extended Data Fig. 12:**
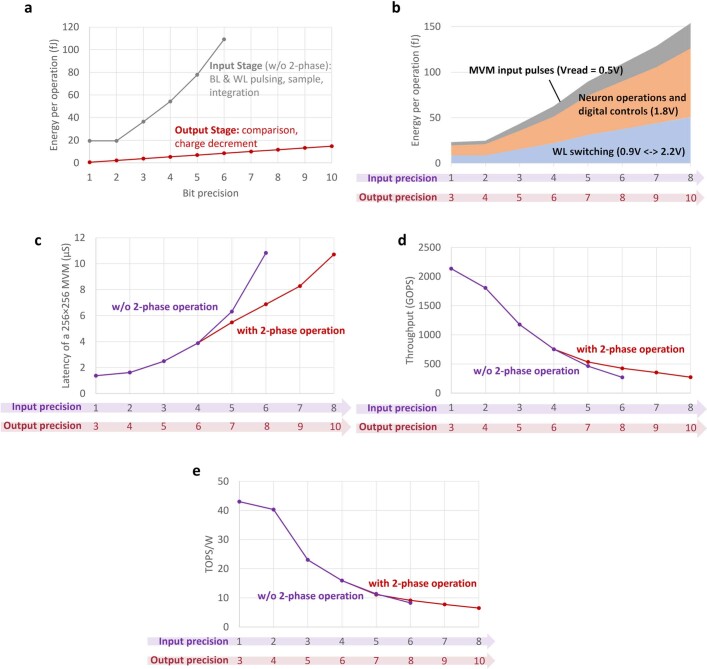
Energy consumption, latency, and throughput measurement results. **a**, Measured energy consumption per operation during the MVM input stage (without 2-phase operation) and output stage, where one multiply–accumulate (MAC) counts as two operations. **b**, Energy consumption breakdown at various MVM input and output bit-precisions. Outputs are 2-bit higher than inputs during a MVM to account for additional precision requirements from partial-sum accumulation. **c**, Latency for performing one MVM with 256×256 weight matrix. **d**, Peak computational throughput (in giga-operations per second). **e**, Throughput-power efficiency (in tera-operations per watt).

Extended Data Fig. 12a shows the energy per operation consumed during the input and the output stages of MVMs under various bit-precisions. The inputs are in the signed integer format, where the first bit represents the sign, and the other bits represent the magnitude. One-bit (binary) and two-bit (ternary) show similar energy because each input wire is driven to one of three voltage levels. Binary input is therefore just a special case for ternary input. It is noted that the curve shown in Extended Data Fig. 12a is obtained without the two-phase operation. As a result, we see a super-linear increase of energy as input bit-precision increases. Similar to the inputs, the outputs are also represented in the signed integer format. The output-stage energy consumption grows linearly with output bit-precision because one additional binary search cycle is needed for every additional bit. The output stage consumes less energy than the input stage because it does not involve toggling highly capacitive WLs that are driven at a higher voltage, as we discuss below.

For the MVM measurements shown in Extended Data Fig. 12b–e, the MVM output stage is assumed to use 2-bit-higher precision than inputs to account for the additional bit-precision required for partial-sum accumulations. The required partial-sum bit-precision for the voltage-mode sensing implemented by NeuRRAM is much lower than that required by the conventional current-mode sensing. As explained before, conventional current-sensing designs can only activate a fraction of rows each cycle, and therefore need many partial-sum accumulation steps to complete an MVM. In contrast, the proposed voltage-sensing scheme can activate all the 256 input wires in a single cycle, and therefore requires less partial-sum accumulation steps and lower partial-sum precisions.

Extended Data Fig. 12b shows the energy consumption breakdown. A large fraction of energy is spent in switching on and off the WLs that connect to gates of select transistors of RRAM devices. These transistors use thick-oxide I/O transistors to withstand high-voltage during RRAM forming and programming. They are sized large enough (width 1 µm and length 500 nm) to provide sufficient current for RRAM programming. As a result, they require high operating voltages and add large capacitance to the WLs, both contributing to high power consumption (*P* = *fCV*^2^, where *f* is the frequency at which the capacitance is charged and discharged). Simulation shows that each of the 256 access transistors contributes about 1.5 fF to a WL; WL drivers combined contribute about 48 fF to each WL; additional WL capacitance is mostly from the inter-wire capacitance from neighbouring BLs and WLs. The WL energy is expected to decrease significantly if RRAMs can be written by a lower voltage and have a lower conductance state, and if a smaller transistor with better drivability can be used.

For applications that require probabilistic sampling, the two counter-propagating LFSR chains generate random Bernoulli noises and inject the noises as voltage pulses into neurons. We measure each noise-injection step to consume on average 121 fJ per neuron, or 0.95 fJ per weight, which is small compared with other sources of energy consumption shown in Extended Data Fig. 12b.

Extended Data Fig. 12c–e shows the measured latency, peak throughput and throughput-power efficiency for performing the 256 × 256 MVMs. It is noted that we used EDP as a figure of merit for comparing designs rather than throughput-power efficiency as tera-operations per second per watt (TOPS W^−1^, reciprocal of energy per operation), because it captures the time-to-solution aspect in addition to energy consumption. Similar to previous work in this field, the reported throughput and energy efficiency represent their peak values when the CIM array utilization is 100%, and does not include time and energy spent at buffering and moving intermediate data. Future work that integrates intermediate data buffers, partial-sum accumulators and so on within a single complete CIM chip should show energy efficiency measured on end-to-end AI applications.

### Projection of NeuRRAM energy efficiency with technology scaling

The current NeuRRAM chip is fabricated using a 130-nm CMOS technology. We expect the energy efficiency to improve with the technology scaling. Importantly, isolated scaling of CMOS transistors and interconnects is not sufficient for the overall energy-efficiency improvement. RRAM device characteristics must be optimized jointly with CMOS. The current RRAM array density under a 1T1R configuration is limited not by the fabrication process but by the RRAM write current and voltage. The current NeuRRAM chip uses large thick-oxide I/O transistors as the ‘T’ to withstand >4-V RRAM forming voltage and provide enough write current. Only if we lower both the forming voltage and the write current can we obtain higher density and therefore lower parasitic capacitance for improved energy efficiency.

Assuming that RRAM devices at a newer technology node can be programmed at a logic-compatible voltage level, and the required write current can be reduced such that the size of the connecting transistor keeps shrinking, the EDP improvements will come from (1) lower operating voltage and (2) smaller wire and transistor capacitance, that is, Energy ∝ *CV*^2^ and Delay ∝ *CV/I*. At 7 nm, for instance, we expect the WL switching energy (Extended Data Fig. 12b) to reduce by about 22.4 times, including 2.6 times from WL voltage scaling (1.3 V → 0.8 V), and 8.5 times from capacitance scaling (capacitance from select transistors, WL drivers and wires are all assumed to scale with minimum metal pitch 340 nm → 40 nm). Peripheral circuit energy (dominated by the neuron readout process) is projected to reduce by 42 times, including 5 times from VDD scaling (1.8 V → 0.8 V) and 8.5 times from smaller parasitic capacitance. The energy consumed by the MVM pulses and charge transfer process is independent of the range of RRAM conductance, as power consumption and settling time of the RRAM array scale with the same conductance factor that cancels in their product. Specifically the energy per RRAM MAC is *E*_MAC_ = *C*_par_ var(*V*_in_), limited only by the parasitic capacitance per unit RRAM cell *C*_par_, and the variance in the driven input voltage var(*V*_in_). Therefore, the MVM energy consumption will reduce by approximately 34 times, including 4 times from read-voltage scaling (0.5 V → 0.25 V), and 8.5 times from smaller parasitic capacitance. Overall, we expect an energy consumption reduction of about 34 times when scaling the design from 130 nm to 7 nm.

In terms of the latency, the current design is limited by the long integration time of neuron, caused primarily by the relatively large integration capacitor size (104 fF), which was chosen conservatively to ensure function correctness and testing different neuron operating conditions. At more advanced technology nodes, one could use a much smaller capacitor size to achieve a higher speed. The main concern for scaling-down capacitor size is that the fabrication-induced capacitor size mismatch will take up a higher fraction of total capacitance, resulting in a lower SNR. However, previous ADC designs have used a unit capacitor size as small as 50 aF (ref. ^56^; 340 times smaller than our *C*_sample_). For a more conservative design, a study has shown that in a 32-nm process, a 0.45-fF unit capacitor has only 1.2% average standard deviation^57^. Besides, the integration time also depends on the drive current of the transistors. Assuming that the transistor current density (μA μm^−1^) stays relatively unchanged after VDD scaling, and that the transistor width in the neuron scales with the contact gate pitch (310 nm → 57 nm), the total transistor drive current will reduce by 5.4 times. As a result, when scaling *C*_sample_ from 17 fF to 0.2 fF and *C*_integ_ proportionally from 104 fF to 1.22 fF, the latency will improve by 15.7 times. Therefore, conservatively, we expect the overall EDP to improve by at least 535 times when scaling the design from 130-nm to 7-nm technology. Extended Data Table 2 shows that such scaling will enable NeuRRAM to deliver higher energy and area efficiency than today’s state-of-the-art edge inference accelerators^58–61^.

## Online content

Any methods, additional references, Nature Research reporting summaries, source data, extended data, supplementary information, acknowledgements, peer review information; details of author contributions and competing interests; and statements of data and code availability are available at 10.1038/s41586-022-04992-8.

## Figures and Tables

**Extended Data Table 1:**
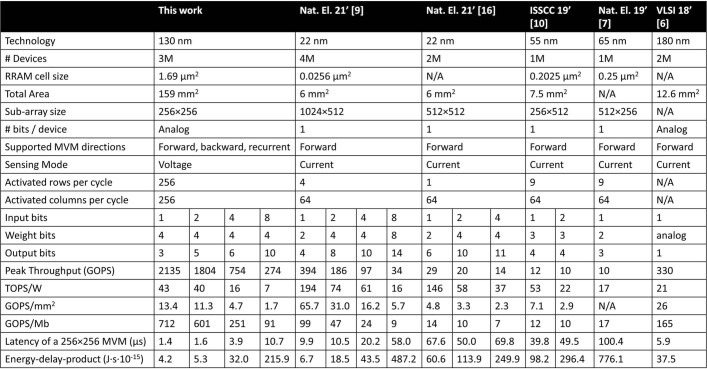
Comparison of fully integrated RRAM-based CIM hardware

Notes:

1. The table does not include studies that do not report detailed performance metrics.

2. All the reported metrics are measured for performing 256×256 matrix-vector multiplications.

3. All reported numbers represent peak throughput/efficiency when array utilization is 100%.

**Extended Data Table 2:**
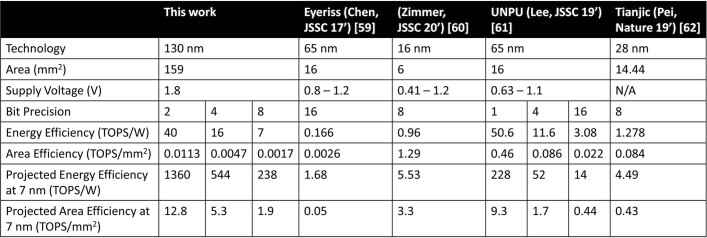
Comparison with digital CMOS AI inference accelerators

Notes:

1. The method to project NeuRRAM efficiency to 7 nm is explained in Methods.

2. The energy-efficiency projection of digital accelerators is based on CV^2^ scaling, where C scales with minimum metal pitch and V is adjusted to nominal VDD of 7 nm.

3. The area-efficiency projection of digital accelerators is based on minimum metal pitch scaling along both horizontal and vertical directions.

## Data Availability

The datasets used for benchmarks are publicly available^18–20^. Other data that support the findings of this study are available in a public repository^47^.

## Code availability

The software toolchain used to test and deploy AI tasks on the NeuRRAM chip, and the codes used to perform noise-resilient model training and chip-in-the-loop progressive model fine-tuning are available in a public repository^47^.

## References

[1] Wong HSP (2012). Metal-oxide RRAM. Proc. IEEE.

[2] Prezioso M (2015). Training and operation of an integrated neuromorphic network based on metal-oxide memristors. Nature.

[3] Ambrogio S (2018). Equivalent-accuracy accelerated neural-network training using analogue memory. Nature.

[4] Ielmini D, Wong HSP (2018). In-memory computing with resistive switching devices. Nat. Electron..

[5] Yao P (2020). Fully hardware-implemented memristor convolutional neural network. Nature.

[6] Mochida, R. et al. A 4M synapses integrated analog ReRAM based 66.5 TOPS/W neural-network processor with cell current controlled writing and flexible network architecture. In *Symposium on VLSI Technology*, *Digest of Technical Papers* 175–176 (IEEE, 2018).

[7] Chen WH (2019). CMOS-integrated memristive non-volatile computing-in-memory for AI edge processors. Nat. Electron..

[8] Khaddam-Aljameh, R. et al. HERMES core-A 14nm CMOS and PCM-based in-memory compute core using an array of 300ps/LSB linearized CCO-based ADCs and local digital processing. In *IEEE Symposium on VLSI Circuits, Digest of Technical Papers* JFS2-5 (IEEE, 2021).

[9] Hung JM (2021). A four-megabit compute-in-memory macro with eight-bit precision based on CMOS and resistive random-access memory for AI edge devices. Nat. Electron..

[10] Xue, C. X. et al. A 1Mb multibit ReRAM computing-in-memory macro with 14.6ns parallel MAC computing time for CNN based AI edge processors. In *IEEE International Solid-State Circuits Conference (ISSCC), Digest of Technical Papers* 388–390 (IEEE, 2019).

[11] Cai F (2019). A fully integrated reprogrammable memristor–CMOS system for efficient multiply–accumulate operations. Nat. Electron..

[12] Ishii, M. et al. On-chip trainable 1.4M 6T2R PCM synaptic array with 1.6K stochastic LIF neurons for spiking RBM. In *International Electron Devices Meeting (IEDM), Technical Digest* 14.2.1–14.2.4 (IEEE, 2019).

[13] Yan, B. et al. RRAM-based spiking nonvolatile computing-in-memory processing engine with precision-configurable in situ nonlinear activation. In *Symposium on VLSI Technology*, *Digest of Technical Papers* T86–T87 (IEEE, 2019).

[14] Wan, W. et al. A 74 TMACS/W CMOS-RRAM neurosynaptic core with dynamically reconfigurable dataflow and in-situ transposable weights for probabilistic graphical models. In *IEEE International Solid-State Circuits Conference (ISSCC)*, *Digest of Technical Papers* 498–500 (IEEE, 2020).

[15] Liu, Q. et al. A fully integrated analog ReRAM based 78.4TOPS/W compute-in-memory chip with fully parallel MAC computing. In *IEEE International Solid-State Circuits Conference (ISSCC), Digest of Technical Papers* 500–502 (IEEE, 2020).

[16] Xue CX (2021). A CMOS-integrated compute-in-memory macro based on resistive random-access memory for AI edge devices. Nat. Electron..

[17] Narayanan P (2021). Fully on-chip MAC at 14 nm enabled by accurate row-wise programming of PCM-based weights and parallel vector-transport in duration-format. IEEE Trans. Electron Devices.

[18] LeCun Y, Bottou L, Bengio Y, Haffner P (1998). Gradient-based learning applied to document recognition. Proc. IEEE.

[19] Krizhevsky, A. & Hinton, G. *Learning Multiple Layers of Features from Tiny Images* (2009); https://www.cs.toronto.edu/~kriz/learning-features-2009-TR.pdf

[20] Warden, P. Speech commands: a dataset for limited-vocabulary speech recognition. Preprint at https://arxiv.org/abs/1804.03209 (2018).

[21] Joshi, V. et al. Accurate deep neural network inference using computational phase-change memory. *Nat. Commun.* **11**, 2473 (2020). 10.1038/s41467-020-16108-9 PMC7235046 32424184

[22] Alibart F, Zamanidoost E, Strukov DB (2013). Pattern classification by memristive crossbar circuits using ex situ and in situ training. Nat. Commun..

[23] Eryilmaz, S. B. et al. Experimental demonstration of array-level learning with phase change synaptic devices. In *International Electron Devices Meeting (IEDM)*, *Technical Digest* 25.5.1–25.5.4 (IEEE, 2013).

[24] Burr GW (2015). Experimental demonstration and tolerancing of a large-scale neural network (165 000 synapses) using phase-change memory as the synaptic weight element. IEEE Trans. Electron Devices.

[25] Eryilmaz SB (2016). Training a probabilistic graphical model with resistive switching electronic synapses. IEEE Trans. Electron Devices.

[26] Sheridan PM (2017). Sparse coding with memristor networks. Nat. Nanotechnol..

[27] Yao P (2017). Face classification using electronic synapses. Nat. Commun..

[28] Banbury, C. et al. MLPerf tiny benchmark. In *Conference on Neural Information Processing Systems (NeurIPS) Track on Datasets and Benchmarks* (2021).

[29] Roy S, Sridharan S, Jain S, Raghunathan A (2021). TxSim: modeling training of deep neural networks on resistive crossbar systems. IEEE Trans. Very Large Scale Integr. Syst..

[30] Yang, T. J. & Sze, V. Design considerations for efficient deep neural networks on processing-in-memory accelerators. In *International Electron Devices Meeting (IEDM)*, *Technical Digest* 22.1.1–22.1.4 (IEEE, 2019).

[31] Lecun Y, Bengio Y, Hinton G (2015). Deep learning. Nature.

[32] Hochreiter S, Schmidhuber J (1997). Long short-term memory. Neural Comput..

[33] Koller, D. & Friedman, N. *Probabilistic Graphical Models: Principles and Techniques (Adaptive Computation and Machine Learning series)* (MIT Press, 2009).

[34] Su, J. W. et al. A 28nm 64Kb inference-training two-way transpose multibit 6T SRAM compute-in-memory macro for AI edge chips. In *IEEE International Solid-State Circuits Conference (ISSCC), Digest of Technical Papers* 240–242 (IEEE, 2020).

[35] Guo, R. et al. A 5.1pJ/neuron 127.3us/inference RNN-based speech recognition processor using 16 computing-in-memory SRAM macros in 65nm CMOS. In *IEEE Symposium on VLSI Circuits, Digest of Technical Papers* 120–121 (IEEE, 2019).

[36] Wang Z (2018). Fully memristive neural networks for pattern classification with unsupervised learning. Nat. Electron..

[37] Shafiee, A. et al. ISAAC: a convolutional neural network accelerator with in-situ analog arithmetic in crossbars. In *Proc. 2016 43rd International Symposium on Computer**Architecture (ISCA)* 14-26 (IEEE/ACM, 2016).

[38] Ankit, A. et al. PUMA: a programmable ultra-efficient memristor-based accelerator for machine learning inference. In *International Conference on Architectural Support for Programming Languages and Operating Systems (ASPLOS*) 715–731 (ACM, 2019).

[39] Wan, W. et al. A voltage-mode sensing scheme with differential-row weight mapping for energy-efficient RRAM-based in-memory computing. In *Symposium on VLSI Technology, Digest of Technical Papers* (IEEE, 2020).

[40] Murmann, B. Digitally assisted data converter design. In *European Conference on Solid-State Circuits (ESSCIRC)* 24–31 (IEEE, 2013).

[41] Zhao, M. et al. Investigation of statistical retention of filamentary analog RRAM for neuromophic computing. In *International Electron Devices Meeting (IEDM), Technical Digest* 39.4.1–39.4.4 (IEEE, 2018).

[42] Alibart F, Gao L, Hoskins BD, Strukov DB (2012). High precision tuning of state for memristive devices by adaptable variation-tolerant algorithm. Nanotechnology.

[43] He, K., Zhang, X., Ren, S. & Sun, J. Deep residual learning for image recognition. In *Proc. IEEE Computer Society Conference on Computer Vision and Pattern Recognition (CVPR)* 770–778 (IEEE, 2016).

[44] Cauwenberghs, G. & Bayoumi, M. A. *Learning on Silicon—Adaptive VLSI Neural Systems* (Kluwer Academic, 1999).

[45] Hornik, K., Stinchcombe, M. & White, H. Multilayer feedforward networks are universal approximators. *Neural Netw.* **2**, 359–366 (1989).

[46] Choi, J. et al. PACT: parameterized clipping activation for quantized neural networks. Preprint at https://arxiv.org/abs/1805.06085 (2018).

[47] Wan, W. weierwan/Neurram_48core: Initial Release (Version 1.0) [Computer software]. *Zenodo*10.5281/zenodo.6558399 (2022).

[48] Jung S (2022). A crossbar array of magnetoresistive memory devices for in-memory computing. Nature.

[49] Jerry, M. et al. Ferroelectric FET analog synapse for acceleration of deep neural network training. In *International Electron Devices Meeting* (*IEDM), Technical Digest* 6.2.1–6.2.4 (IEEE, 2018).

[50] Jiang Z (2019). Next-generation ultrahigh-density 3-D vertical resistive switching memory (VRSM)–Part II: design guidelines for device, array, and architecture. IEEE Trans. Electron Devices.

[51] Cauwenberghs G (1996). An analog VLSI recurrent neural network learning a continuous-time trajectory. IEEE Trans. Neural Netw..

[52] Wu, W. et al. A methodology to improve linearity of analog RRAM for neuromorphic computing. In *Symposium on VLSI Technology, Digest of Technical Papers* 103–104 (IEEE, 2018).

[53] Ji, Y. et al. FPSA: a full system stack solution for reconfigurable ReRAM-based NN accelerator architecture. In *International Conference on Architectural Support for Programming Languages and Operating Systems (ASPLOS)* 733–747 (ACM, 2019).

[54] Esser, S. K., Mckinstry, J. L., Bablani, D., Appuswamy, R. & Modha, D. S. Learned step size quantization. In *International Conference on Learning Representations (ICLR)* (2020).

[55] Jung, S. et al. Learning to quantize deep networks by optimizing quantization intervals with task loss. In *IEEE/CVF Conference on Computer Vision and Pattern Recognition (CVPR)* 4345–4354 (IEEE/CVF, 2019).

[56] Stepanovic D, Nikolic B (2013). A 2.8 GS/s 44.6 mW time-interleaved ADC achieving 50.9 dB SNDR and 3 dB effective resolution bandwidth of 1.5 GHz in 65 nm CMOS. IEEE J. Solid State Circuits.

[57] Tripathi V, Murmann B (2014). Mismatch characterization of small metal fringe capacitors. IEEE Trans. Circuits Syst. I Regul. Pap..

[58] Chen YH, Krishna T, Emer JS, Sze V (2017). Eyeriss: an energy-efficient reconfigurable accelerator for deep convolutional neural networks. IEEE J. Solid State Circuits.

[59] Zimmer B (2020). A 0.32-128 TOPS, scalable multi-chip-module-based deep neural network inference accelerator with ground-referenced signaling in 16 nm. IEEE J. Solid State Circuits.

[60] Lee J (2019). UNPU: an energy-efficient deep neural network accelerator with fully variable weight bit precision. IEEE J. Solid State Circuits.

[61] Pei J (2019). Towards artificial general intelligence with hybrid Tianjic chip architecture. Nature.

[62] Murmann, B. *ADC Performance Survey 1997–2021* (2021); https://web.stanford.edu/~murmann/adcsurvey.html

